# Hub-organized parallel circuits of central circadian pacemaker neurons for visual photoentrainment in *Drosophila*

**DOI:** 10.1038/s41467-018-06506-5

**Published:** 2018-10-12

**Authors:** Meng-Tong Li, Li-Hui Cao, Na Xiao, Min Tang, Bowen Deng, Tian Yang, Taishi Yoshii, Dong-Gen Luo

**Affiliations:** 1 State Key Laboratory of Membrane Biology, College of Life Sciences, Peking University, 100871 Beijing, China; 2 IDG/McGovern Institute for Brain Research, Peking University, 100871 Beijing, China; 3 Peking-Tsinghua Center for Life Sciences, Academy for Advanced Interdisciplinary Studies, Peking University, 100871 Beijing, China; 4 PTN Graduate Program, College of Life Sciences, Peking University, 100871 Beijing, China; 5 Center for Quantitative Biology, Academy for Advanced Interdisciplinary Studies, Peking University, 100871 Beijing, China; 6 Graduate School of Natural Science and Technology, Okayama University, Okayama, 700-8530 Japan

## Abstract

Circadian rhythms are orchestrated by a master clock that emerges from a network of circadian pacemaker neurons. The master clock is synchronized to external light/dark cycles through photoentrainment, but the circuit mechanisms underlying visual photoentrainment remain largely unknown. Here, we report that *Drosophila* has eye-mediated photoentrainment via a parallel pacemaker neuron organization. Patch-clamp recordings of central circadian pacemaker neurons reveal that light excites most of them independently of one another. We also show that light-responding pacemaker neurons send their dendrites to a neuropil called accessary medulla (aMe), where they make monosynaptic connections with Hofbauer–Buchner eyelet photoreceptors and interneurons that transmit compound-eye signals. Laser ablation of aMe and eye removal both abolish light responses of circadian pacemaker neurons, revealing aMe as a hub to channel eye inputs to central circadian clock. Taken together, we demonstrate that the central clock receives eye inputs via hub-organized parallel circuits in *Drosophila*.

## Abstract (web summary)

The central circadian clock in *Drosophila* is made up of ~ 150 anatomically distributed neurons; the circuits underlying photoentrainment is unclear. This study describes ex vivo patch-clamp recording of the eye-mediated light response of all known circadian clock neurons, and shows that they are organized in parallel circuits centered around a hub.

## Introduction

Daily rhythms of physiology and behavior are orchestrated by a master circadian clock in the brain. To properly adapt the physiology and behavior to the time of day, the master clock must be reset every day via the environmental cues such as the light/dark (LD) cycle^1–3^. Chronic misalignment between the master clock and the external environment has been implicated in pathological symptoms, such as sleep disorders, impaired cognition, and even cancer^4,5^. Although synchronization or entrainment of the master clock to external LD cycles has been intensely studied, the precise circuit mechanisms underlying visual photoentrainment remain incompletely understood^2,6–10^.

In mammals, the master clock locates in the suprachiasmatic nuclei (SCN) of hypothalamus^2^. A hierarchical circuit organization has been proposed for the photoentrainment of the SCN master clock^8,11–13^, in which the ventrolateral SCN neurons receive light inputs from the retina and then relay the light information to entrain the dorsomedial SCN neurons. Constructing the neural circuit of the master clock for photoentrainment requires the identification of all light-responding pacemaker neurons and their light-input pathways.

In contrast to the daunting number of SCN pacemaker neurons (up to 20,000 in mice), the central clock of *Drosophila* is composed of 150 well-defined circadian pacemaker neurons that distribute across the brain, including three groups of dorsal neurons (DN1–3) and three groups of lateral neurons (LNv, LNd, and LPN)^14–17^. The LNv neurons are further grouped into small LNvs (s-LNvs) and large LNvs (l-LNvs). The six dorsal lateral neurons (LNd) are distinguished by their neuropeptides, with one expressing ion transport peptide (ITP) and two expressing short neuropeptide F (sNPF)^18^. Thus, *Drosophila* is an ideal model for elucidating the neural circuits for photoentrainment of the central clock, with a small number of central circadian pacemaker neurons, clear pacemaker neuron identification, and powerful genetic tools^3,7,19–21^.

In *Drosophila*, circadian photoreception is mediated by both visual systems and the circadian pacemaker neurons in a cell-autonomous manner^1,22–25^. Cryptochrome (CRY), a blue-light-sensitive protein that express in many of the 150 central circadian pacemaker neurons, is responsible for the cell-autonomous photoentrainment in *Drosophila*^1,22–25^. It has been well established that upon light stimulation cryptochrome interacts with and then degrades the TIMELESS protein to reset the molecular clock^26-28^. Interestingly, cryptochrome was also reported to be a blue-light sensor that increases neuronal firing in the l-LNvs^29^, but its physiological functions and contributions to circadian photoentrainment remain unclear^10,30^. Recently, the cryptochrome-mediated electrical responses to light was implicated in the arousal behaviors of flies^31^. However, in the absence of cryptochrome, *Drosophila* can still entrain to the LD cycle^1,22–25^, implying the flies also entrain via light inputs from visual systems. For eye-driven photoentrainment, a model with a hierarchical circadian pacemaker neuron organization has also been widely proposed^10,30,32,33^, similar to that of mammals^8,11–13^. That is, the LNvs are thought to receive eye inputs and then relay light information to synchronize the rest central circadian pacemaker neurons. Specifically, after receiving eye inputs, s-LNvs transmit light information to entrain DNs and LNds by releasing neuropeptide pigment-dispersing factor (PDF) in the dorsal brain. In addition, l-LNvs receive eye inputs and then relay light signals to s-LNvs and LNds by releasing PDF in the accessory medulla (aMe)^10,16,32–36^. However, the light-induced electrical responses of the 150 central circadian pacemaker neurons and the circuits mediating light inputs from the eyes to central circadian pacemaker neurons remain largely unknown.

Here, by achieving patch-clamp recordings from each of the entire group of central circadian pacemaker neurons in *Drosophila*, we discover that most of them are excited to fire action potentials by light inputs through the eyes. Notably, these eye-mediated light responses of most central circadian pacemaker neurons remain intact when other circadian pacemaker neurons are silenced or ablated. Each of these light-responding central circadian pacemaker neurons sends its dendrites to the aMe to receive light inputs directly from visual systems. Combining circuit tracing, laser ablation, opto/chemogenetics, multi-electrode patch-clamp recordings, and behavioral studies, we map out the circuit connectivity between visual systems and central circadian pacemaker neurons in *Drosophila*. This functional connectome reveals that the central clock receives visual inputs via the hub-organized parallel circuits of central circadian pacemaker neurons.

## Results

### Photoentrainment by subgroups of circadian pacemaker neurons

**Fig. 1:**
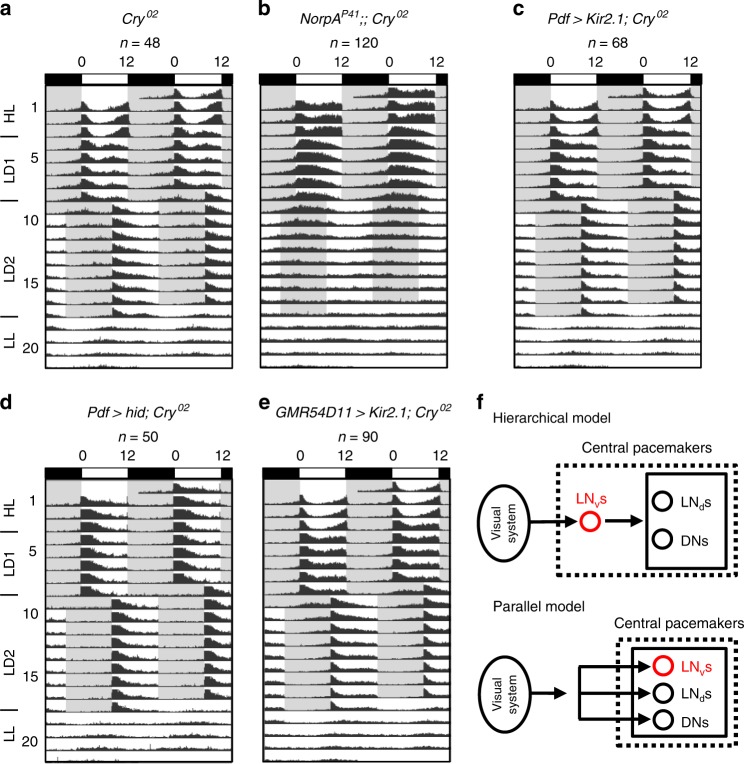
Non-hierarchical photoentrainment by central pacemaker neurons. **a** Averaged actogram shows the *Cry*^*02*^ flies entrain to dim LD cycles. The bars above actograms indicate light conditions: white is lights-ON and black is lights-OFF. Note the advance of evening peaks in *Cry*^*02*^ flies after switching from the HL (~200 lux) to dim LD1 (~0.05 lux) cycles and the slow re-entrainment of evening peaks under the dim LD2 (~0.05 lux) cycles with an 8-h phase delay from LD1. *n* is the number of flies averaged in each group. **b** The entrainment is lost in *NorpA*^*P41*^;;*Cry*^*02*^ flies. **c** *Cry*^*02*^ flies entrain to dim LD cycles with PDF-expressing LNvs silenced by *Kir2.1*. **d** *Cry*^*02*^ flies entrain to dim LD cycles with PDF-expressing LNvs ablated. **e** *Cry*^*02*^ flies entrain to dim LD cycles with the fifth s-LNv and ITP-LNd silenced by *Kir2.1*. **f** Two alternative models of the circuitry organizations of pacemaker neurons to receive visual signals for photoentrainment. Top, the hierarchical model; bottom, the non-hierarchical or parallel model

Consistent with previous findings^22,37^, the CRY knockout *Cry*^*02*^ flies were able to re-entrain to new LD cycles under dim light conditions (Fig. 1a) and the entrainment was eliminated in *Cry*^*02*^ and *NorpA*^*P41*^ double-mutant flies (Fig. 1b) that also lacked the phospholipase C (PLC)-mediated phototransduction in the eyes^38^, revealing the eye-mediated photoentrainment. Interestingly, the evening peak of *Cry*^*02*^ mutant flies showed an immediate advance after the flies were released from high light (HL) into the dim LD cycles, which then slowly entrained during the dim LD cycles. This result is consistent with the idea that light can deaccelerate the evening oscillators^39^, thus producing an apparent advance when switching HL to dim LD cycles. To investigate whether circadian rhythms were reset by visual inputs through the hierarchical circadian pacemaker neuron circuits as generally thought^10,30,32,33^, we silenced/killed certain groups of central circadian pacemaker neurons to keep only part of the circadian pacemaker neuron circuits functional in *Cry*^*02*^ flies. Consistent with previous reports by others^40–42^, we found that the *Cry*^*02*^ flies maintained an ability to photoentrain via visual inputs from the eyes when the PDF-expressing LNvs were silenced by *Kir2.1* (Fig. 1c) or even killed by the expression of a pro-death protein, hid (Fig. 1d), further confirmed by the phase difference between the flies entrained with and without the 8-h phase-delayed LD2 cycles (Supplementary Fig. 1). In addition, we found that silencing the evening pacemaker neurons of the fifth s-LNv and ITP-LNd that were labeled by *GMR54D11-Gal4*^43^ did not eliminate photoentrainment in *Cry*^*02*^ flies (Fig. 1e and Supplementary Fig. 1). Under this condition, there was still an evening peak because the driver of *GMR54D11-Gal4* targeted only two evening neurons, the fifth s-LNv and ITP-LNd, both of which produced robust electrical responses to light stimuli and could also communicate their visual responses to other LNds (see results below). These data are consistent with the idea^34^ that, in the absence of cryptochrome, either LNvs or LNds were able to photoentrain the flies via visual inputs. Thus, the central pacemaker neurons might be organized in a parallel rather than a hierarchical way to receive eye inputs for visual photoentrainment (Fig. 1f).

### Widespread of light responses in circadian pacemaker neurons

**Fig. 2:**
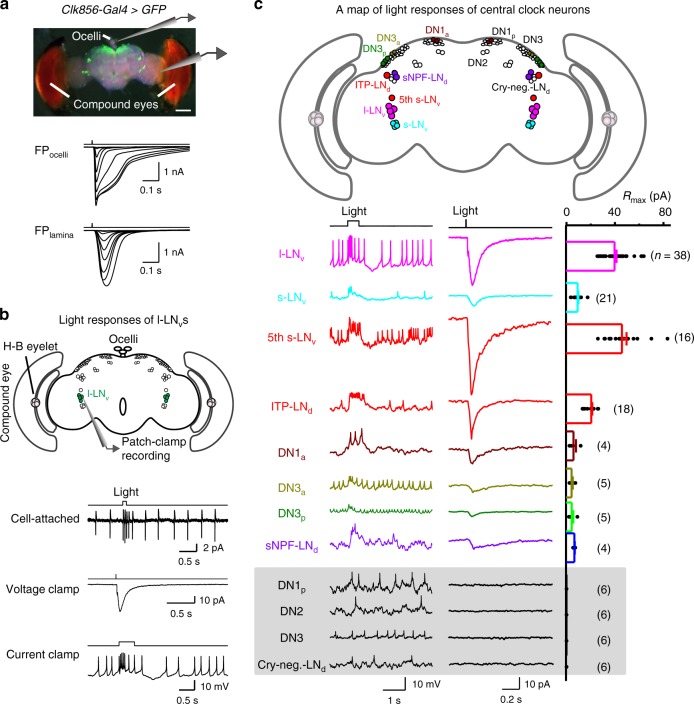
Light-induced responses are widespread in pacemaker neurons. **a** A brain preparation for recording light responses of central pacemaker neurons. Top, overlay of bright field and GFP images of a brain preparation. Scale bar, 100 μm. Middle, families of superimposed field potential of ocelli (FP_ocelli_), bottom, field potential recorded in the lamina (FP_lamina_). Light stimulation: 2 ms, 470 nm. The timing of flashes is indicated on top of the response traces. **b** Light-induced electrical responses of the l-LNvs. Top, illustration of patch-clamp recordings on the l-LNvs. Bottom three traces are light responses in the cell-attached, voltage clamp (perforated patch-clamp recording), and current clamp (perforated patch-clamp recording) configurations, respectively. Light stimulation: 470 nm. **c** A map of light responses across pacemaker neurons. Top, schematic distribution of pacemaker neurons. Light-sensitive pacemaker neuron groups are color-coded, but labeled only in the left hemisphere for clarity, and light-insensitive pacemaker neurons are labeled only in the right hemisphere. Bottom, light-induced spike firing (current clamp, left), current responses (voltage clamp, middle), and collective data of the maximal current responses (right) of individual pacemaker neurons. DN3a and DN3p cells are recorded in the presence of picrotoxin. Data are represented as average ± SEM. *n* indicates the cell numbers

To investigate the neural basis underlying the above behavioral results, we first examined whether and how central pacemaker neurons responded to light stimulation by patch-clamp recordings. We developed an ex vivo fly brain preparation that maintains all eye structures intact (Fig. 2a). In this preparation, the ocelli and compound eyes maintain their light sensitivity. The central pacemaker neurons were identified with an array of *Gal4* and *LexA* drivers^21,43^ (see also Methods and Supplementary Table 1). In cell-attached recordings, the l-LNvs responded to full field light by firing a train of action potentials (Fig. 2b). Correspondingly, light triggered an inward current that depolarized the l-LNvs in perforated patch-clamp recordings. Likewise, the morning anticipatory s-LNvs^44,45^ also responded to light with an increase of action-potential firing (Fig. 2c).

Next, we examined the evening pacemaker neurons and found that the fifth s-LNv produced an inward current to increase action-potential firing upon light stimulation (Fig. 2c). Similarly, the ITP-LNd also responded to light with an increase in action-potential firing (Fig. 2c). Therefore, both the morning- and evening-anticipatory pacemaker neurons responded to light stimulation in a similar manner by increasing the firing frequency of action potentials.

A systematic examination of the entire population of pacemaker neurons further revealed that the DN1a, DN3a, DN3p (three large posterior DN3 cells), and sNPF-LNd neurons responded to light stimulation (Fig. 2c), but most of the DN1p, DN2, DN3 (not including the DN3p cells), and cryptochrome-negative LNd cells did not show obvious electrical responses to light stimulations (Supplementary Fig. 2). These results provide the first comprehensive map of light-responding pacemaker neurons across the entire central clock in *Drosophila* brain.

We then investigated the origin of the light responses of these pacemaker neurons. There are seven eyes in *Drosophila*, including a pair of compound eyes, two Hofbauer–Buchner (H–B) eyelets, and three ocelli^46^. To dissect out the contribution of individual eyes, we first physically removed the three ocelli from the ex vivo brain preparation and found that light responses of pacemaker neurons were not changed (Supplementary Fig. 3a). In contrast, light responses were abolished by the removal of the compound eyes (Supplementary Fig. 3b). Removing the compound eyes also destroys the H–B eyelets because the latter are physically connected to the compound eyes^47^. This result demonstrated that light-induced electrical responses of central circadian pacemaker neurons are mainly driven by visual inputs from external eyes under our experimental conditions, but not by light-induced cell-autonomous electrical responses as reported by others^29^ (see Discussion).

In *Drosophila*, phototransduction in the eyes is mediated by a PLC-mediated cascade^48^. PLC mutant *NorpA*^*P24*^ flies^49^ do not exhibit light responses in their eye photoreceptors. We generated flies with the *NorpA*^*P24*^; *DvPdf-Gal4*, *UAS-mCD8-GFP* genotype, which allowed us to examine the impact of the loss of canonical phototransduction on circadian pacemaker neurons. Notably, light responses were completely eliminated in the l-LNv, s-LNv, fifth s-LNv, ITP-LNd pacemaker neurons (Supplementary Fig. 3c). Light responses of circadian pacemaker neurons were also eliminated in another PLC mutant^38^, the *NorpA*^*P41*^ fly (Supplementary Fig. 3d). On the other hand, the light responses of central circadian pacemaker neurons remained intact when CRY was knocked out (Supplementary Fig. 3d). Therefore, the light-induced electrical responses of circadian pacemaker neurons are driven by the PLC-mediated canonical phototransduction in the compound eyes, H–B eyelets, or both but not the ocelli, consistent with a recent behavioral study^37^.

### Circadian pacemaker neurons receive eye inputs independently

**Fig. 3:**
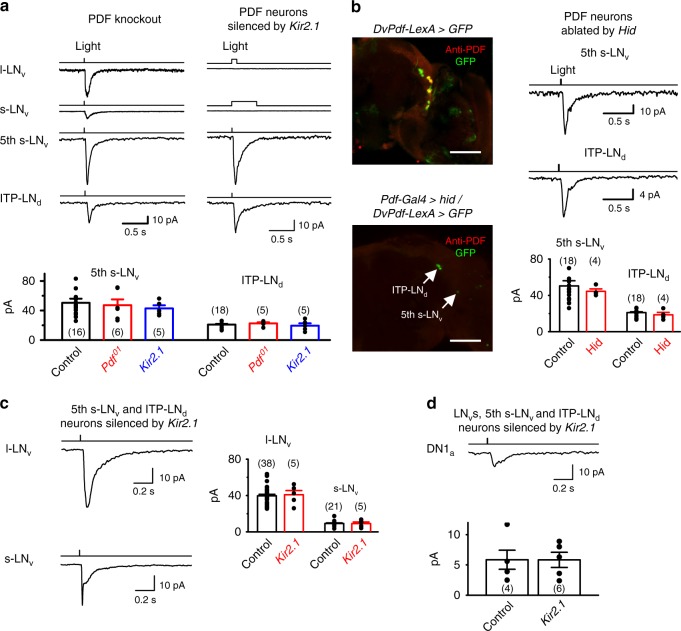
Central pacemaker neurons receive visual signals in a parallel manner. **a** PDF is not required for light responses of pacemaker neurons. Top, responses of pacemaker neurons in *Pdf*^*01*^ flies (left) and in flies with the PDF-expressing LNvs silenced by *Kir2.1* (right). Under cell-attached recordings, the s-LNvs and l-LNvs show neither spontaneous nor light-induced action-potential firing (right, top two traces). Light-induced inward currents of fifth s-LNv and ITP-LNd cells (right, bottom two traces, voltage clamp recordings). Light stimulation: 470 nm. Bottom, collective data of the fifth s-LNv and ITP-LNd responses in flies as indicated. The responses are not significantly different among the fifth s-LNvs or ITP-LNds under different conditions. **b** PDF-expressing LNvs are not required for light responses of evening pacemaker neurons. Left, overlay of PDF immunostaining and GFP fluorescence in control flies (top) and in flies with PDF-expressing LNv ablated (bottom). Scale bar, 50 μm. Right, light responses of the fifth s-LNv and ITP-LNd cells (top) in flies with PDF-expressing LNvs ablated and collective data (bottom). The responses are not significantly different among the fifth s-LNvs or ITP-LNds. **c** Left, light responses of l-LNvs (top) and s-LNvs (bottom) in flies with evening oscillators silenced by *Kir2.1*. Right, collective data. The responses are not significantly different among the l-LNvs or s-LNvs. **d** Top, light responses of DN1a in flies with the PDF-expressing LNvs, fifth s-LNv, and ITP-LNd silenced by *Kir2.1*. Bottom, collective data. The responses are not significantly different between the control and *Kir2.1* groups. Cell numbers are indicated in brackets

Next, we examined whether the light responses of evening circadian pacemaker neurons were reduced or abolished in the absence of PDF, as predicted by the hierarchical circadian pacemaker neuron photoentrainment model^10,30,32,33^. To test this idea, we examined the light-induced responses of evening circadian pacemaker neurons in the PDF knockout *pdf*^*01*^ flies or in the PDF receptor mutant *Han*^*5304*^ flies. Surprisingly, we found that the fifth s-LNv and ITP-LNd cells still showed robust light responses in *Pdf*^*01*^ (Fig. 3a) and *Han*^5304^ (Supplementary Fig. 4a), indicating that the evening circadian pacemaker neurons responded to light independently of PDF signaling. Consistent with this idea, the light responses remained intact in evening circadian pacemaker neurons when the PDF-expressing LNvs were silenced by *Kir2.1* (Fig. 3a and Supplementary Fig. 4b, c). Next, we selectively ablated these LNvs by expressing *hid* with *Pdf-Gal4*, confirmed with PDF-antibody immunostaining (Fig. 3b). Surprisingly, we found that the fifth s-LNv and ITP-LNd cells still showed robust light-induced responses in the absence of LNvs (Fig. 3b). Similarly, the PDF-expressing LNvs still responded to light when the fifth s-LNv and ITP-LNd neurons were silenced (Fig. 3c). In addition, DN1a cells still responded to light when the PDF-expressing LNvs, fifth s-LNvs, and ITP-LNd were all silenced (Fig. 3d). Therefore, in contrast to the commonly held view of a hierarchical architecture, the central circadian pacemaker neurons received the eye-mediated light information independently of one another.

### Accessary medulla channels eye inputs to pacemaker neurons

**Fig. 4:**
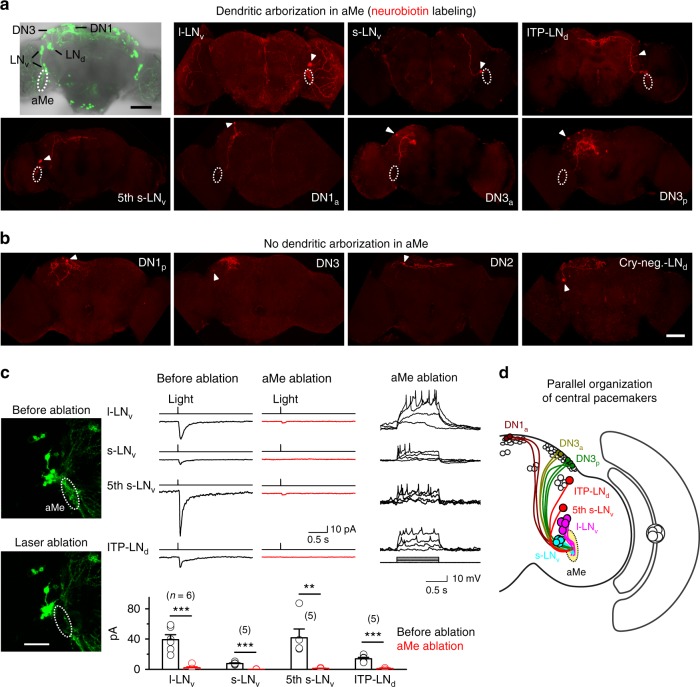
aMe channels visual inputs to central pacemaker neurons. **a** aMe arborization revealed by single-cell neurobiotin labeling. Overlay of DIC and GFP images shows the aMe and pacemaker neurons (top, left panel). The other panels show neurobiotin immunostaining of individual pacemaker neurons, with cell bodies marked by arrowheads. Dashed circles indicate the aMe. Scale bar, 50 μm. **b** Light-insensitive pacemaker neurons (DN1p, DN2, DN3, and Cry-negative LNd cells) do not send any processes to the aMe. Cell bodies are indicated by arrowheads. Scale bar: 50 μm. **c** aMe is required for light responses of pacemaker neurons. Left: laser ablation of the aMe, with images before (top) and after (bottom) ablation. Dashed lines indicate the aMe. Laser: 800 nm, 50 mW. Scale bar, 50 μm. Middle, light responses of the same l-LNv, s-LNv, fifth s-LNv, and ITP-LNd cells before and after the aMe ablation (top) and the average results (bottom). Right, pacemaker neurons are able to be electrically excited in flies with the aMe ablated. Current steps: 2 pA. All of the eight repeated experiments show similar results. *n* = 6, 5, 5, and 5 for l-LNv, s-LNv, fifth s-LNv, and ITP-LNd, respectively; error bars represent SEM; **P < 0.01; ***P < 0.001. **d** Schematic illustration of independent visual inputs to the pacemaker neurons

To investigate the neural mechanisms underlying independent light receiving by central circadian pacemaker neurons, we examined their anatomy to reconstruct the circuit connectivity. GFP expression with specific drivers has revealed many fundamental characteristics of circadian pacemaker neurons^14–17^, but the GFP signals could be confounded by the overlap of neuronal clusters. To achieve a single-cell resolution of the anatomy, we used neurobiotin-labeling technique by injecting the dye into each individual circadian pacemaker neuron through a patch-clamp recording pipette^50^. With this fine tool, we found that the l-LNv, s-LNv, fifth s-LNv, ITP-LNd, DN1a, DN3a, and DN3p cells, all of which being light responsive, sent dendrites to a neuropil, the accessary medulla (aMe) (Fig. 4a and Supplementary Fig. 5a), consistent with prior findings^14–17^. By contrast, the light-insensitive DN1p, DN2, DN3, and cryptochrome-negative LNd cells did not send any neuronal processes to the aMe (Fig. 4b).

The dendritic arborization in the aMe suggested that these central circadian pacemaker neurons might receive visual inputs from the aMe. To test this idea, we performed selective laser ablation of the aMe. Guided by the GFP expression with *DvPdf-Gal4*, we used an 800-nm laser to ablate the aMe and found that the l-LNv, s-LNv, fifth s-LNv, and ITP-LNd pacemaker neurons lost their light responses but maintain electrical excitability (Fig. 4c). Therefore, we demonstrated that the aMe acted as a hub that channeled eye inputs to the central circadian pacemaker neurons (Fig. 4d).

### sNPF-LNds receive inputs from the fifth s-LNv and ITP-LNd cells

**Fig. 5:**
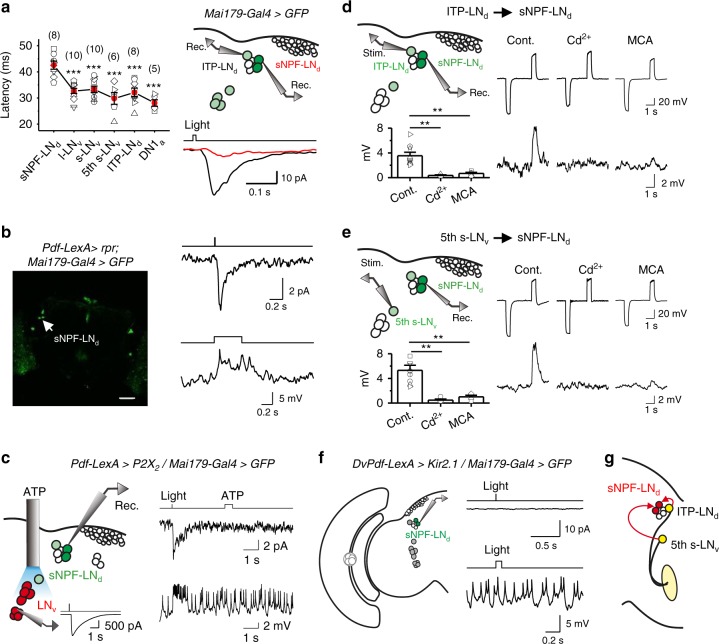
sNPF-LNds receive inputs from the fifth s-LNv and ITP-LNd cells. **a** sNPF-LNd responses have long latency. Left, average data. Right, dual patch-clamp recordings on pairs of sNPF-LNd and ITP-LNd (top) and their light responses (bottom). Light stimulation: 470 nm. Cell numbers are indicated in brackets; error bars represent SEM; ***P < 0.001 (comparison with sNPF-LNd). **b** PDF-expressing LNvs are not required for light responses of sNPF-LNds. Left, the LNvs are ablated by *reaper*. Scale bar: 50 μm. Right, light responses (top, voltage clamp; bottom, current clamp) of sNPF-LNds remain intact in the absence of LNvs. The average response of 6.2 ± 0.8 pA (*n* = 6, mean ± SEM) is not significantly different from the WT control of 6.6 ± 0.4 pA (*n* = 4). **c** PDF-expressing LNvs do not excite sNPF-LNds. Left, scheme of simultaneous LNv activation and sNPF-LNd recordings. The LNvs expressing *P2X*_*2*_ are activated by ATP, with a representative s-LNv response shown in the inset. Right, sNPF-LNds respond to light but not to LNv activation by ATP. ATP: 2.5 mM, 500 ms; light: 470 nm, 2 ms. The bar trace indicates the timing of ATP and light stimulations. Three out of three sNPF-LNd recordings show no ATP responses. **d** sNPF-LNds receive inputs from the ITP-LNd. Left, scheme of dual patch-clamp recordings (top) and average sNPF-LNd responses (bottom). Right, ITP-LNd stimulation triggers sNPF-LNd responses. *n* = 9, 4, and 4 for control, 100 μM Cd^2+^, and 50 μM MCA, respectively; error bars represent SEM; **P < 0.01. **e** sNPF-LNds receive inputs from the fifth s-LNv. Left, scheme of dual patch-clamp recordings (top) and average sNPF-LNd responses (bottom). Right, fifth s-LNv stimulation triggers sNPF-LNd responses. *n* = 8, 4, and 4 for control, 100 μM Cd^2+^, and 50 μM MCA, respectively; error bars represent SEM; **P < 0.01. **f** The fifth s-LNv and ITP-LNd are required for sNPF-LNd light responses. Left, illustration of sNPF-LNd recordings when the fifth s-LNv and ITP-LNd are silenced by *Kir2.1*. Right, current (top) and voltage (bottom) responses of sNPF-LNds to light stimulation. Five out of five recordings show no light responses. **g** Schematic illustration of visual inputs from the fifth s-LNv and ITP-LNd to sNPF-LNds

Interestingly, the light-sensitive sNPF-LNds did not send neuronal processes to the aMe, but their responses were abolished by aMe ablation (Supplementary Fig. 6). In addition, their response latency was longer than that of other circadian pacemaker neurons and even the simultaneously recorded ITP-LNd in the same preparation (Fig. 5a), indicating that they received visual inputs from other light-sensitive pacemaker neurons but not directly from the aMe. However, selectively killing the PDF-expressing LNvs did not affect the light responses of sNPF-LNds (Fig. 5b), implying that the PDF-expressing LNvs did not provide visual inputs to the sNPF-LNds. Consistently, ATP activation of the ATP-gated *P2X*_*2*_ channel^51^-expressing LNvs did not elicit any electrical responses in the sNPF-LNds (Fig. 5c).

Next, we investigated whether the sNPF-LNds received inputs from the evening circadian pacemaker neurons that showed large light-induced electrical responses. For this purpose, we performed dual patch-clamp recordings on pairs of sNPF-LNd and ITP-LNd cells. Depolarizing the ITP-LNd elicited a depolarization in the sNPF-LNd, but hyperpolarizing the ITP-LNd could not elicit any responses of the sNPF-LNd (Fig. 5d), suggesting the involvement of excitatory chemical synaptic transmission from the ITP-LNd to sNPF-LNds. Consistently, we found that both Cd^2+^ and a nicotinic acetylcholine receptor antagonist, mecamylamine (MCA), blocked the transmission from the ITP-LNd to sNPF-LNds (Fig. 5d). In addition, we also found a Cd^2+^/MCA-sensitive chemical synaptic transmission from the fifth s-LNv to sNPF-LNds (Fig. 5e). Finally, silencing the fifth s-LNv, ITP-LNd, and PDF-expressing LNvs abolished the light responses of sNPF-LNds (Fig. 5f). These data demonstrated that the sNPF-LNds received visual inputs from both the fifth s-LNv and ITP-LNd (Fig. 5g), consistent with the idea that circadian pacemaker neurons communicate with each other^43,52,53^.

### Monosynaptic inputs from H–B eyelets to pacemaker neurons

**Fig. 6:**
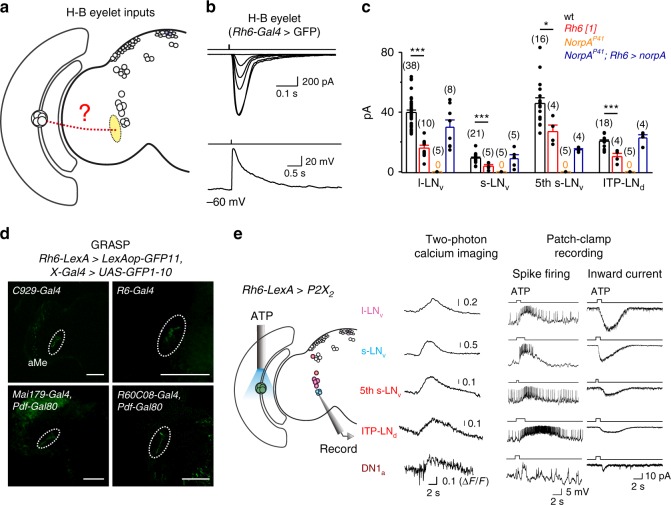
Monosynaptic visual inputs from H–B eyelets to pacemaker neurons. **a** Schematic illustration of H–B eyelet inputs to the central pacemaker neurons. **b** Response properties of H–B eyelet photoreceptors. Top, light elicits inward currents that produce graded depolarization of H–B eyelet photoreceptors. Bottom, no spike firing in H–B eyelet photoreceptors to light-induced depolarization under current clamp. **c** Collective data of light responses in pacemaker neurons of the different flies as indicated. 0 indicates no responses at all. Cell numbers are indicated in brackets; error bars represent SEM; *P < 0.05; ***P < 0.001. **d** Close contacts between H–B eyelet photoreceptors and different pacemaker neurons revealed by GRASP. The aMe is marked by dashed circles. Scale bar: 50 μm. **e** H–B eyelets excite pacemaker neurons. Left, illustration of H–B eyelet activation by ATP. Middle, two-photon GCaMP6m imaging of pacemaker neurons in response to the ATP activation of H–B eyelets. Right, electrical responses of pacemaker neurons. ATP: 2.5 mM

The eye removal experiments (Supplementary Fig. 3) suggested that the central pacemaker neurons received visual inputs from H–B eyelets or compound eyes, or both. Next, we investigated whether the H–B eyelets provided visual inputs to central circadian pacemaker neurons (Fig. 6a). There are four photoreceptors in each H–B eyelet^47^. However, the nature of their light responses is unknown. We performed patch-clamp recordings on the eyelet photoreceptors and found that a brief flash of light triggered an inward current that depolarized the eyelet photoreceptor (Fig. 6b). Like the retinal photoreceptors in compound eyes, H–B eyelet photoreceptors did not fire action potentials. Interestingly, H–B eyelet was reported to be able to use a PLC-independent light signaling to photoentrain the flies^49^. To examine the phototransduction mechanisms in H–B eyelet photoreceptors, we next performed patch-clamp recordings on the eyelet photoreceptors of *norpA* mutant flies and found a complete loss of light-induced electrical responses (Supplementary Fig. 7a). In addition, we found that H–B eyelet photoreceptors lost their light-induced electrical responses in Rhodopsin 6 (*Rh6)* mutant flies (Supplementary Fig. 7a). These data demonstrated that H–B eyelet photoreceptors detect light with Rh6 through the PLC-mediated phototransduction. This conclusion was further supported by our finding that light-induced electrical responses were restored to the H–B eyelet photoreceptors after rescuing *norpA* by *Rh6-Gal4* in *norpA* mutant flies (Supplementary Fig. 7b). The light responses of central circadian pacemaker neurons in *Rh6[1]* flies, which lacked Rh6, were dramatically reduced (Fig. 6c), revealing visual inputs from H–B eyelets. This conclusion was supported by a partial restoration of light responses of circadian pacemaker neurons in *NorpA*^*P41*^ flies when light signaling of the H–B eyelets was restored by rescuing their PLC with *Rh6-Gal4* (Fig. 6c). Furthermore, the light-induced electrical responses of l-LNvs and s-LNvs that were mediated by Rh6 visual pigments in *Rh6-Gal4*-driven *norpA* rescued flies were largely eliminated after laser ablation of the axons of H–B eyelet photoreceptors (Supplementary Fig. 8).

We then reconstructed the circuit linking H–B eyelets to the central circadian pacemaker neurons. Consistent with prior studies^33,47,49,54^, our single-cell neurobiotin labeling showed that the H–B eyelet photoreceptors projected their axons to the aMe (Supplementary Fig. 5b and Supplementary Fig. 7c), where the dendrites of light-sensitive circadian pacemaker neurons arborized. To examine whether H–B eyelet photoreceptors made synapses with all of the circadian pacemaker neurons that had dendritic arborization in the aMe, we performed GFP reconstitution across synaptic partners (GRASP)^55^. We used the *Rh6-LexA* driver for the eyelets and corresponding drivers for the s-LNv (*R6-Gal4*), l-LNv (*C929-Gal4*), fifth s-LNv, and ITP-LNd (*Mai179-Gal4* and *Pdf-Gal80*), and DN1a cells (*GMR60C08-Gal4* and *Pdf-Gal80*; see also Supplementary Fig. 9). The GRASP signals appeared in the aMe, indicating close contacts between the eyelet photoreceptors and these individual groups of pacemaker neurons (Fig. 6d). To test whether these contacts represented functional synapses, we recorded the neuronal activities of these circadian pacemaker neurons to the activation of H–B eyelet photoreceptors. We found that the ATP activation of the *P2X*_*2*_-expressing H–B eyelets increased genetically encoded calcium indicator (GCaMP6m) fluorescence and action-potential firing of the s-LNv, l-LNv, fifth s-LNv, ITP-LNd, and DN1a cells (Fig. 6e and Supplementary Fig. 10a, b). To rule out the potential confounding effects of activating Rh6-expressing R8 photoreceptors^56^ by diffusible ATP, we performed two-photon optogenetic activation of the aMe, where R8 photoreceptors have no direct reach, with *CsChrimson* expression by *Rh6-Gal4*. We observed similar post-synaptic responses in the l-LNvs (Supplementary Fig. 7d). On the other hand, a recent study reported excitatory inputs from H–B eyelet to s-LNvs but not to l-LNvs^33^, which might be due to its usage of a less sensitive method based on calcium imaging with GCaMP3. Therefore, the H–B eyelet photoreceptors send their light information to central circadian pacemaker neurons via monosynaptic transmission.

### *AstC/CcapR* interneurons relay compound-eye inputs to the clock

**Fig. 7:**
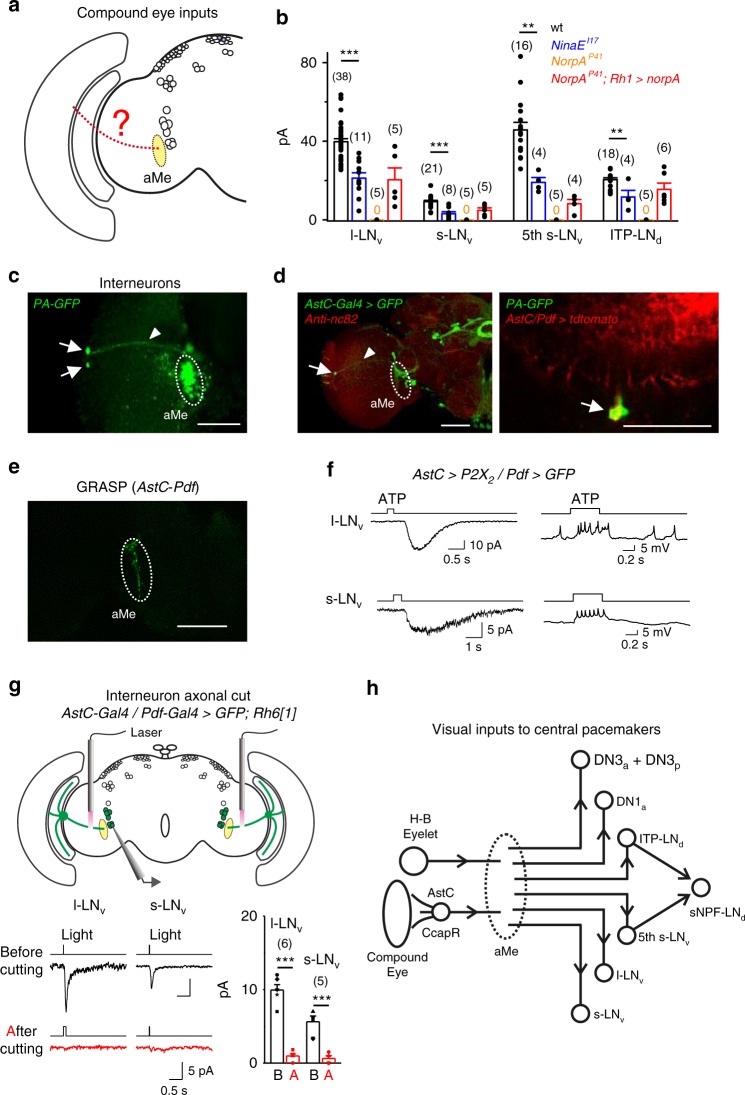
*AstC/CcapR* interneurons relay compound-eye inputs to the clock. **a** Schematic illustration of compound-eye inputs to the central pacemaker neurons. **b** Collective data of light responses in pacemaker neurons of the different flies as indicated. Cell numbers are indicated in brackets; error bars represent SEM; **P < 0.01; ***P < 0.001. **c** Interneurons connecting compound eyes to pacemaker neurons. Two interneurons (marked by arrows) are PA-GFP-labeled after two-photon photoactivation of the aMe. **d** *AstC* interneuron labels one PA-GFP interneurons. Left, *AstC-Gal4* labels one interneuron with similar anatomical features as the PA-GFP-labeled interneurons. Right, overlay of the *AstC* and PA-GFP labeling. Dashed circles indicate the aMe, arrows indicate the cell bodies, and arrowheads indicate the axons. Scale bar: 50 μm. **e** GRASP signals between *AstC* interneuron and PDF-expressing LNvs. Dashed circle indicates the aMe. Scale bar: 50 μm. **f** *AstC* neuron excites pacemaker neurons. Post-synaptic responses of the l-LNvs (top two traces) and s-LNvs (bottom two traces) to the ATP activation of *P2X*_*2*_-expressing *AstC* neuron. ATP: 2.5 mM. **g** *AstC/CcapR* interneurons are required for visual inputs from compound eyes to pacemaker neurons. Top, schematic illustration of axonal cutting by laser and simultaneously patch-clamp recordings of pacemaker neurons. Bottom, light responses of l-LNvs and s-LNvs before and after laser cut of the *AstC/CcapR* axons (left) and the average results (right). *n* = 6 and 5 for l-LNv and s-LNv, respectively; error bars represent SEM; ***P < 0.001. **h** A schematic summary of the circuit connectivity between visual system and central pacemaker neurons

To examine whether compound eyes contribute to light responses of central circadian pacemaker neurons (Fig. 7a), we recorded central circadian pacemaker neurons in *NinaE*^*I17*^ flies that lack rhodopsin 1 (Rh1) and found their light responses were significantly reduced compared to wild-type flies (Fig. 7b). Therefore, the compound eyes provided light information to central circadian pacemaker neurons, further supported by the partial restoration of light-induced responses in central circadian pacemaker neurons of *NorpA*^*P41*^ flies when the PLC was rescued back to the compound eyes with *Rh1-Gal4* (Fig. 7b).

Retinal photoreceptors of compound eyes terminate in the lamina or medulla^56^, but not in the aMe where the dendrites of central circadian pacemaker neurons arborize. We speculate that the light signals from compound eyes to central circadian pacemaker neurons are mediated by visual interneurons in the optic lobe. We searched for the potential interneurons using photoactivatable GFP (PA-GFP)^57^. We expressed PA-GFP pan-neuronally with the *GMR57C10-LexA* driver and used two-photon microscopy to activate PA-GFP in the aMe, where the axons of potential interneurons should project. We consistently found two PA-GFP-labeled interneurons with cell bodies located between the lamina and medulla (Fig. 7c). To obtain the driver lines that specifically label these two interneurons, we screened hundreds of *Gal4* and *LexA* drivers that captured the expression of individual neurotransmitters and neuropeptides and their receptors (generated by Dr. Yi Rao’s lab). We uncovered that allatostatin C (*AstC*)*-Gal4* labeled one PA-GFP interneuron (Fig. 7d) and crustacean cardioactive peptide receptor (*CcapR*)-*Gal4* labeled both of the two PA-GFP interneurons (Supplementary Fig. 11a). The *AstC*-labeled interneuron overlapped with one of the *CcapR* interneurons (Supplementary Fig. 11b). Consistently, single-cell neurobiotin injection to the *AstC* neuron revealed that its axon targeted to the aMe (Supplementary Fig. 5b and Supplementary Fig. 11c). Intersection between serotonin (5-HT) and *AstC/CcapR* labeled these interneurons more specifically, revealing their intensive arborization in the lamina (Supplementary Fig. 11d). These anatomical data indicated that *AstC*/*CcapR* interneurons might relay visual signals from compound eyes to central circadian pacemaker neurons.

Robust GRASP signals between the *AstC* neuron and LNvs were detected in the aMe (Fig. 7e) indicating synaptic contacts between them. Consistently, ATP activation of the *P2X*_*2*_-expressing *AstC* neuron increased action-potential firing of s-LNvs and l-LNvs (Fig. 7f and Supplementary Fig. 10c, d). On the other hand, selective laser ablation of the axons of *AstC*/*CcapR* interneurons significantly reduced the light responses of the LNvs mediated by compound eyes (Fig. 7g). Furthermore, we found that the *AstC* interneuron generated graded depolarization to light stimulation (Supplementary Fig. 12a). Like many other visual interneurons^58^, it did not fire action potentials (Supplementary Fig. 12b). Therefore, the *AstC* interneuron relayed light signals from the compound eyes to central circadian pacemaker neurons, as did the *CcapR*-expressing interneurons (Supplementary Fig. 13). Taken together, our results revealed an unexpected circuit organization of light inputs to central circadian pacemaker neurons, in which the light signals from H–B eyelets and compound eyes were channeled to the central pacemaker neurons via the aMe (Fig. 7h).

## Discussion

The master circadian clock in the brain is a critical time-keeping mechanism. Over the past decades, seminal discoveries have been made in understanding the molecular and behavioral clocks. By contrast, how central circadian pacemaker neurons of the master clock are organized to produce coherent and adaptive circadian rhythms in response to external inputs remain poorly understood. Here, we reconstruct the circuit connectivity between visual systems and central circadian pacemaker neurons in *Drosophila*. This functional connectome reveals that the central circadian pacemaker neurons are organized in parallel with one another to receive eye inputs, thus producing robust and efficient photoentrainment.

Circadian clocks show intrinsic rhythms that do not exactly equal 24 h. To adapt to the 24-h day, the circadian clocks need to be reset daily. Light is the primary factor that resets the clock. In mammals, a complete entrainment to an 8-h phase delay takes 1 week. In contrast, *Drosophila* is entrained to the same phase delay within 1 day. The fast photoentrainment in *Drosophila* is mainly mediated by cryptochrome in a cell-autonomous manner^1,22,23^. Light-activated cryptochrome degrades the TIMELESS protein to reset the molecular clock. In the absence of cryptochrome, photoentrainment still occurs, though at a much slower speed. The slow entrainment is mediated by light inputs from the eyes, which resembles the mammalian entrainment^2,59,60^.

Central clock entrainment by eye inputs is generally proposed to be composed of two hierarchical circuits^2,8,10,32^. That is, a recipient circuit of a key group of circadian pacemaker neurons receives inputs from visual systems. A synchronizing circuit then communicates light information from the recipient circadian pacemaker neurons to the rest circadian pacemaker neurons within the central clock. Strikingly, our results revealed that most central circadian pacemaker neurons in *Drosophila* receive the eye-mediated light signal independently of one another, thus simultaneously effecting the two circuit functions of light receiving and synchronization.

We found that the central circadian pacemaker neurons receive visual inputs through hub-organized parallel, instead of hierarchical, circuits based on five experimental findings. First, most central circadian pacemaker neurons, including the l-LNv, s-LNv, fifth s-LNv, ITP-LNd, DN1a, DN3a, and DN3p cells, were excited to fire action potentials by light. Second, light responses of these circadian pacemaker neurons did not depend on cryptochrome or PDF. Third, laser ablation of the aMe or eye removal eliminated light responses of circadian pacemaker neurons. Fourth, light-responding l-LNv, s-LNv, fifth s-LNv, ITP-LNd, DN1a, and DN3a cells sent their dendrites to the aMe to receive inputs from H–B eyelets and indirectly from compound eyes. Fifth, selectively silencing either the LNvs or LNds in *Cry*^*02*^ flies did not eliminate photoentrainment to the dim LD cycle. How does the light-induced electrical responses of central pacemaker neurons entrain circadian rhythms? Recently, it has been shown that action-potential firing in central circadian pacemaker neurons can produce phase shifting^32,36^, which exploits the E3 ligase component CUL-3 to degrade TIMELESS protein^32^.

In addition to receiving visual inputs from the eyes, many central clock neurons in *Drosophila* express the deep-brain photoreceptor cryptochrome, which can reset the molecular clock in response to blue light^1,22–25^. Prior work has shown that l-LNvs respond to intense and long-duration blue-light stimulations by increasing action-potential firing in an ex vivo brain preparation with external eyes attached^61^. The light-induced responses were absent in *Cry*^*01*^ or *Cry*^*02*^ null mutant flies^29^, but unaffected in *glass*^*60j*^ flies that lacked external eye photoreceptors^29^. Those results indicate that l-LNvs exhibit light-induced electrical responses that are dependent on cryptochrome and independent of the input from the external eyes. Here, we examined light-induced electrical responses systematically in all clock neurons with an ex vivo brain preparation that maintained seven external eyes, including two compound eyes, three ocelli, and two H–B eyelets. We found that many clock neurons, including l-LNvs, showed excitatory responses to brief light stimuli (Fig. 2); these responses did not depend on cryptochrome function (Supplementary Fig. 3c). In contrast, virtually all light responses that we measured throughout the pacemaker network (including those of l-LNvs) were absent when external eye input was removed either physically (Supplementary Fig. 3b and 3d) or genetically (Supplementary Fig. 3c and 3d). These independent surgical and genetic results clearly demonstrate that, under our recording conditions, light responses of clock neurons were mediated mainly by external eye photoreceptors, with little to no contribution by cryptochrome.

The discrepancy between our observations of only eye-mediated electrical responses to light and prior observations^29^ of only cryptochrome-mediated responses to light in the l-LNvs cannot be easily explained. It likely reflects one or more fundamental technical differences. To promote future studies that may address these differences, we propose two methodological issues as possible sources for the discrepant results: (i) the details of the dissection methods and (ii) the intensity/duration parameters of the light stimuli. Regarding (i) dissection methods: in our experience, the intactness of the eye structures, optic nerve, and the neuropils of optic lobes was a critical factor to ensure that we could observe the eye-mediated light responses in l-LNvs and other clock neurons. Hence the addition of a digestive enzyme such as papain to loosen cell interactions (thus also likely compromise those synaptic connections that mediate inputs from external eyes to clock neurons) throughout the dissection used by Fogle et al.^29,61^ may be here a relevant methodological variable. Regarding (ii) stimulation parameters, we speculate that cryptochrome-mediated electrical responses require light stimuli of much higher intensities and longer durations than those that we employed to study eye-mediated responses. We occasionally obtained small (~1 pA) l-LNv responses in preparations that were surgically disconnected from the external eyes (Supplementary Fig. 14). Importantly, these only occurred when we applied intense and long-duration ultraviolet-blue-light stimuli. In contrast, weak and brief light stimuli reliably generated eye-mediated responses in l-LNvs and other clock neurons in our ex vivo preparations that maintain inputs from the external eyes (Fig. 2).

Unlike the direct inputs from the H–B eyelets, the central circadian pacemaker neurons receive visual inputs from compound eyes via interneurons. Our PA-GFP experiments revealed that *AstC/CcapR*-expressing neurons were among the potential connecting interneurons. This was supported by the GRASP signals between the *AstC* neuron and LNvs. Furthermore, chemogenetic or optogenetic activation of these interneurons could excite the LNvs. On the other hand, laser ablation of the *AstC/CcapR* axons reduced the light responses of the LNvs mediated by compound eyes. Thus, the *AstC/CcapR* interneurons can relay light inputs from compound eyes to central circadian pacemaker neurons, which bypasses the visual circuits in the medulla. This wiring feature is consistent with the role of detecting illumination but not imaging/motion features from visual environments by the central clock.

A similar parallel circadian pacemaker neuron architecture, with different wiring, may also exist for mammalian photoentrainment. In mice, the intrinsically photosensitive retinal ganglion cells (ipRGCs) have been reported to synapse separately with both the ventrolateral and dorsomedial SCN neurons^62^. Therefore, ipRGCs can in principle transmit light information to simultaneously entrain the ventrolateral and dorsomedial SCN neurons. The connectivity difference between mice and *Drosophila* may reflect a functional adaptation. In *Drosophila*, the aMe acts as an analog to digital converter, where light information is transmitted from the non-spiking neurons to the spiking central circadian pacemaker neurons. In mice, ipRGCs are themselves spiking neurons, thus being able to drive circadian pacemaker neurons at a long distance without the need of an equivalent hub structure of aMe. The parallel circuit architecture for central clock photoentrainment represents a remarkable example of evolutionary adaptation to a conserved function across organisms.

## Methods

### Fly stocks

*Clk856-Gal4*, *Cry39-Gal4*, *Han*^*5304*^, and *Pdf*^*01*^ were from Taishi Yoshii. *C929-Gal4*, *Pdf-Gal4* (II and III), *DvPdf-Gal4*, *Mai179-Gal4*, *Cry-Gal80*, *NorpA*^*P41*^, *Cry*^*01*^, and *Cry*^*02*^ were from Patrick Emery. *R6-Gal4*was from Paul Taghert. *Pdf-LexA* and* Clk4.1M-Gal4* were from Amita Sehgal. *Pdf-Gal80*, *LexAop-mCD4::spGFP11*, *UAS-mCD4::spGFP1–10*, *UAS-FRT-Stop-FRT-GFP*, and *LexAop2-IVS-SYN21-mSPA-GFP-P10* were from Yi Rao. *Rh6-LexA* was from Takashi Suzuki. *UAS-P2X*_*2*_ (III) was from Zuoren Wang, and *LexAop-P2X*_*2*_ was from Orie Shafer. *NinaE*^*I17*^ (BL5701), *NorpA*^*P24*^, and *Rh1-Gal4* (BL8691) were from Tao Wang. *LexAop2-Kir2.1-GFP* was from Limin Yang. *UAS-Kir2.1* (II and III) were from Chuan Zhou. *UAS-hid* and *UAS-mCD8-GFP* were from Chris Potter. *shakB*^2^ was from Jonathan M. Blagburn. *LexAop-rpr* was from Stephen Cohen. *Rh6-Gal4* (BL7459), *Rh6[1]* (BL109600), *UAS-norpA* (BL26273), *UAS-GCaMP6m* (BL42750), *UAS-CsChrimson* (BL55136), *ninaE*^*I17*^, *Rh6[1]* (BL109601), *LexAop2-GCaMP6m* (BL44588), *LexAop2-GCaMP6f* (BL44277), *LexAop2-IVS-myr::GFP* (BL 32209 and 32210), *UAS-GFP S65T* (BL1522), *UAS-tdtomato* (BL32221 and 32222), *GMR54D11-Gal4* (BL41279), *GMR60C08-Gal4* (BL48227), *GMR57C10-LexA* (BL52817), *Clk9M-Gal4* (BL41810), *UAS-DenMark* (BL33061), and *UAS-syt.eGFP* (BL6926) were from the Bloomington Stock Center. All experimental genotypes used in this study are listed in Supplementary Table 1.

All flies were raised on standard cornmeal agar medium, under 60% humidity and a 12-h light/12-h dark cycle at 25 °C.

### Generation of transgenic flies

To generate the *DvPdf-LexA* line, the 1.9 kb *DvPdf* promoter PCR amplified from *Drosophila Virilis* genomic DNA, with KpnI (5′) and XcmI (3′) sites added to the primers. Primer sequences: forward: 5′–TACGAAGTTATGCTAGCGGAGGTACCAGGACGATTCTTGACCG–3′; reverse: 5′–TCGCTTCTTCTTGGGTGGCATGGTGGCCACTTGAAACTTGGGAATGAACA–3′. This promoter fragment was inserted into pUASTattB and the *SV40-LexA* was inserted into downstream of the *DvPdf* promoter.

To generate *AstC-Gal4* and *CcapR-Gal4* flies, coding sequence of *Gal4* is inserted into AstC (CG14919) and CcapR (CG33344) loci just before the stop codon by homologous recombination. To generate *Trh-FLP* flies, coding sequence of flippase is inserted into Trh (CG9122) locus before the stop codon by homologous recombination.

### Electrophysiological recordings

Dissections and recordings were performed in a dark room. Young adult flies (1–4 days after eclosion) with correct genotypes were selected randomly regardless of gender and then immobilized on ice. The head was dissected and transferred to a recording chamber, with its proboscis, antenna, and cuticle removed by fine forceps. The compound eyes and ocelli were kept intact, and the brain preparation was stabilized with grease, anterior side up. To record H–B eyelet photoreceptors and DN3p, the posterior side was positioned to face up. The brain preparation was continuously perfused with a saline solution bubbled with 95% O_2_/5% CO_2_ (~pH 7.3) at room temperature. The saline is composed of (in mM): 103 NaCl, 3 KCl, 4 MgCl_2_, 1.5 CaCl_2_, 26 NaHCO_3_, 1 NaH_2_PO_4_, 5-*N*-tri-(hydroxymethyl)-methyl-2-aminoethane-sulfonic acid (TES), 20 d-glucose, 17 sucrose, and 5 trehalose. The osmolarity of the saline is 280 mOsm. Recordings were performed at Zeitgeber time (ZT)2–5, ZT12–15, and ZT20–23, and no difference was observed for light-induced responses at different ZT time. The dissection was made under dim red light to minimize light activation. Before recordings, the brain preparation was kept in the recording chamber in darkness for ~5 min.

The targeted neurons were identified by GFP fluorescence with a ×60 water-immersion objective (Nikon) and visualized for patch-clamp recordings with infrared-differential interference contrast (IR-DIC) optics on multiphoton microscope (Nikon, A1 MP). The image was displayed on a monitor (Sony) through an IR-CCD (DAGE-MTI). To access clock neurons in the deeper brain, a focal and brief application of protease XIV (0.67 mg ml^−1^; Sigma) was used to expose the cell bodies of target neurons. A minimal enzymatic and mechanical treatment was aimed to avoid the circuit damage. Patch-clamp recording electrodes (~15–20 MΩ) were pulled from borosilicate glass (WPI) with a P-1000 puller (Sutter). For whole-cell patch clamp, the recording pipette was filled with internal solution consisting of (in mM): 140 K-gluconate, 6 NaCl, 2 MgCl_2_, 0.1 CaCl_2_, 1 EGTA, 10 HEPES (pH 7.3), with an osmolarity of 270 mOsm. For perforated patch-clamp recording, the pipette was backfilled with the internal solution that contains 150 μg ml^-1 ^amphotericin B, and then filled with regular internal solution. For field potential recording of ocelli and compound eyes, the pipette was filled with the saline solution with NaHCO_3_ replaced by 10 mM HEPES (pH 7.3). For cell-attached recordings, the pipette was also filled with the HEPES saline. All chemicals are from Sigma.

For dual patch-clamp recording, the two targeted neurons were first identified, and then recorded one after another. Current injections (500 ms duration) were repeated every 8 s for 20 trials. Mecamylamine of 50 μM or 100 μM Cd^2+^ was applied by a three-barrel glass tube positioned ~200 μm away from the recorded neurons^63^.

Signals were amplified with MultiClamp 700B (Molecular Devices), digitized with Digidata 1440A (Molecular Devices), recorded with Clampex 10.6 (Molecular Devices), filtered at 2 kHz, and sampled at 5 kHz. The recorded neuron was voltage clamped at −70 mV, otherwise specified. Measured voltages were corrected for a liquid junction potential.

### Pacemaker neuron identification

The identification of pacemaker neurons is based on the GFP expression with specific *Gal4* or *LexA* drivers, the anatomical location, the neurite stratification, and light-induced electrical responses. Specifically, the s-LNvs and l-LNvs are labeled by *R6-Gal4* and *C929-Gal4*, respectively. In addition, these two LNvs can be also labeled by the drivers of *Pdf-Gal4*, *Pdf-LexA*, *Mai179-Gal4*, *DvPdf-Gal4*, and *DvPdf-LexA*. The fifth s-LNv and ITP-LNd are labeled by *GMR54D11-Gal4*^43^, or *Mai179-Gal4* with *Pdf-Gal80*^21^, or *DvPdf-Gal4*^32^, or *DvPdf-LexA*. The sNPF cells are the two smaller cells of the three LNds labeled by *Mai179-Gal4*^21^. The Cry-negative LNds are the three smaller cells of the four LNds labeled by the *DvPdf-Gal4*^32^ or *DvPdf-LexA* drivers. The two DN1a cells are labeled by *Cry39-Gal4*^15^, or *GMR60C08-Gal4*. The two DN2 cells are labeled by the *Clk9M-Gal4*^64^. The two DN3a cells are labeled by the *Cry39-Gal4*^15^. The DN1p cells are labeled by *Clk4.1M-Gal4* or *Clk4.1M-LexA*^65^. The DN3p and other DN3 cells are labeled by *Clk856-Gal4*^66^. *Cry39-Gal4* and *Clk856-Gal4* also label many other pacemaker neurons.

### Light stimulation

The light source to stimulate the brain preparation was a combination of high-power LEDs (LED4D285, LED4D041, M730L4, M405L2, M530L3, and M340L4, Thorlabs). The stimulating light was coupled to the fluorescence port of the A1 MP microscope via a liquid light guide. A reflected filter (MBE49100, Nikon) directed the light through a water-immersion ×60 objective. After recordings for each pacemaker neuron, we confirmed its response properties by delivering light stimuli directly on the ocelli and the ipsilateral compound eye or switching to a ×4 objective to deliver light stimulation to the entire brain preparation. The LEDs were driven by DC4100 (Thorlabs) and the timing and duration of light stimulation was controlled by the analog voltage output of Digidata 1440A. The wavelength was selected via the LED controller, and light intensity was adjusted through the voltage input. The size of illumination spot was measured by a piece of cover glass coated with 480 Alexa dye. It was photobleached for 5 min and imaged the fluorescence for light spot calibration. The spot size was 0.13 mm^2^, covering one-side eye structures of the preparation. Light intensity was daily calibrated with a power meter (Model 1936-R, 918D-ST-UV, Newport).

### Optogenetic stimulation

The flies were fed with foods containing 100 μM all trans-retinal (ATR). A stock solution of 100 mM ATR was first prepared by using alcohol as the solvent. The ATR stock solution was then diluted into the food medium for a final ATR concentration of 100 μM.

For patch-clamp recordings of studying the connections from H–B eyelets to pacemaker neurons, the flies were fed with the ATR food and raised in constant darkness. During the patch recordings, the aMe, where the axons of H–B eyelet photoreceptors target, was stimulated with a laser tuned to 700 nm through a two-photon microscopy.

### Chemogenetic stimulation

ATP-gated ion channel *P2X*_*2*_ were driven by different *Gal4* or *LexA* lines. ATP-Na of 2.5 mM was delivered through a three-barrel tube (~200 μm away from the targeted H–B eyelet), controlled by a stepper (SF77B, Warner Instruments) for rapid solution change^63^.

### Laser ablation of aMe

The aMe structure is identified by GFP expression with *DvPdf-Gal4*. Light responses of the targeted clock neurons were recorded before ablation. The dense neurites of the aMe close to sLNvs were selected as a region of interest (ROI) using the Nikon Nis-Element software. A Maitai DeepSee Ti:Sapphire ultrafast laser (Spectra-physics) tuned to 800 nm was used to ablate the ROI for around 3–5 s with a dwell time of 4.8 μs per pixel. The laser power at the back of the ×60 objective was 50–70 mW at 800 nm. The size, duration, and power of the laser were optimized for different preparations to achieve a visible cavitation bubble and destruction of the aMe. The perforated patch-clamp recordings were maintained during the ablation. After ablation, light response and cell physiology were examined.

### Single-cell labeling by neurobiotin

Two percent neurobiotin (Vector lab, Cat# SP-1120) was dissolved in a modified internal solution (in mM): 70 K-gluconate, 6 NaCl, 2 MgCl_2_, 0.1 CaCl_2_, 1 EGTA, 4 Mg-ATP, 0.5 GTP-Tris, 10 HEPES (pH 7.3), for osmolarity balance of the high concentration of neurobiotin. The recording pipette (20 MΩ) was filled with neurobiotin-containing internal solution. After breaking into whole-cell mode, depolarizing currents (200 ms, 2 Hz) were injected into the cell for 20 min, which facilitated diffusion of positively charged neurobiotin into the recorded neuron. The recording pipette was gently detached from the cell after another 20-min wait of neurobiotin diffusion within the cell. After recordings, the brain preparation was transferred into 4% paraformaldehyde, fixed for 4 h on ice. The brain preparation was then washed by PBS for three times at a 20-min interval, blocked in 5% BSA in PBST (1% Triton in PBS) for 2 h at room temperature. The preparations were incubated with Streptavidin-568 (Invitrogen, Cat#S-11226) overnight at 4 °C, washed by PBST (1%Triton in PBS) for three times at an interval of 20 min, and then mounted in the glycerol. For PDF immunostaining, the brains were first incubated with the mouse PDF antibody (DSHB, Cat#C7, diluted 1:50) overnight at 4 °C. After three-time wash in PBST (1% Triton in PBS), the brains were incubated with Streptavidin-568 and Alexa Fluor 488-conjugated goat anti-mouse antibody (Invitrogen, Cat#A11001, diluted 1:200) overnight at 4 °C. Images were acquired on a Nikon A1R+ confocal microscope with a ×25 water-immersion objective.

### Neuronal tracing with photoactivatable GFP

Photoactivation of PA-GFP was performed to reveal visual inputs to the aMe from compound eyes. The aMe was marked by tdtomato expression with *Pdf-Gal4* and *mSPA-GFP* was pan-neuronally expressed with *GMR57C10-LexA*. We used Nikon A1R+ multiphoton laser scanning microscope to image and photoactivate preparations. After defining the ROI, a laser beam of 720 nm was used for activation with a dwell time of 4.8 μs per pixel for 30 times at intervals of 8 s. Three photoactivation cycles were used at a 1-min interval. After photoactivation, two-photon images with 950 nm were acquired after a 10-min wait for GFP diffusion.

### Synaptic labeling by GRASP and immunostaining

The brains of flies with two split GFP was dissected in the PBS. The brain was transferred into 4% paraformaldehyde for 1 h fixation on ice and then washed by PBS and blocked in 5% normal goat serum in PBST (1% Triton in PBS) for 2 h at room temperature. Subsequently, the brains were incubated with primary antibody mouse anti-GFP (Sigma, Cat#G6539, 1:100) overnight at 4 °C. This antibody is raised against full-length GFP and it is specific for the reconstituted GFP^67^. For standard antibody staining, rabbit anti-PDP1^68^ (1:9000), rabbit anti-GFP (Invitrogen, Cat#A-11122, 1:100), chicken anti-GFP (Rockland, Cat#600-901-215S, 1:1000), mouse anti-PDF (DSHB, Cat#C7, 1:50), and mouse anti-nc82 (DSHB, Cat#nc82, 1:100) were used. After washing with 0.5% PBST three times, the brains were incubated with secondary antibodies, Alexa Fluor 488-conjugated anti-mouse (Invitrogen, Cat#A11001, 1:200), Alexa Fluor 488-conjugated anti-rabbit (Invitrogen, Cat#A11008, 1:200), Alexa Fluor 488-conjugated anti-chicken (Invitrogen, Cat#A11039, 1:200), Alexa Fluor 568-conjugated anti-rabbit (Invitrogen, Cat#11036, 1:200), Alexa Fluor 568-conjugated anti-mouse (Invitrogen, Cat#11004, 1:200), Alexa Fluor 647-conjugated anti-mouse (Invitrogen, Cat#21237, 1:200). The brains were washed three more times before being mounted in glycerol. Images were acquired sequentially in 0.5 μm sections on a Nikon A1R+ confocal microscope with a ×25 water-immersion objective.

### Two-photon-based calcium imaging

Fluorescence was imaged on Nikon A1R+ multiphoton laser scanning microscope, which is equipped with a GaAsP type non-descanned detector (GaAsP-NDD) for high-sensitivity detection of fluorescence signals at 950-nm wavelength, 7 frames per second, 126 × 512 pixels. Patch-clamp recordings were simultaneously performed on the targeted clock neurons. ROIs were selected manually. The fluorescence change was calculated as: Δ*F*/*F* = (*F*_*t*_ − *F*_0_)/*F*_0_ × 100%, where *F*_*t*_ is the fluorescence at time point *t*, and *F*_0_ is the basal fluorescence. ROI fluorescence was subtracted by the background fluorescence.

### Behavioral assays

Flies were raised in 12-h light/12-h dark (12:12 LD). Locomotor activity was assayed with male flies (1–2 days after eclosion) at 21 °C. Individual fly was placed in a glass tube with food and recorded using DAM2 monitors (TriKinetics). Flies were subjected to a 12:12 LD cycle with light intensity of 200 lux for 3 days and the same cycles with light intensity of ~0.05 lux for 5 days, then an 8-h phase-delayed 12:12 LD cycles with light intensity of ~0.05 lux for 9 days, and finally constant dim light of ~0.05 lux till the end of experiments.

Data analysis was done with the actograms using Actogram J^69^. Double-plotted actograms were averaged from the rhythmic flies, and the activity plots were the averaged activity in the first four dim LL cycles across the rhythmic flies.

### Statistics

Sample sizes were determined based on the standard of the field. Data acquisition and analysis were not performed blinded but independently repeated by experimenters. All data were analyzed statistically using one-way analysis of variance, represented as mean ± SEM.

## Electronic supplementary material

Supplementary Information

## Data Availability

All relevant data and code in this study are available from the corresponding author upon reasonable request.
