# Supplementary material for: Hub-organized parallel circuits of central circadian pacemaker neurons for visual photoentrainment in Drosophila
Source: Nat Commun. 2018 Oct 12;9:4247. doi: 10.1038/s41467-018-06506-5 (PMC6185921; doi:10.1038/s41467-018-06506-5)
Supplement: Supplementary file 1 — Supplementary Information [file 41467_2018_6506_MOESM1_ESM.pdf]

Supplementary Information

Hub-organized parallel circuits of central circadian pacemaker neurons for visual photoentrainment in *Drosophila*

Li et al.,

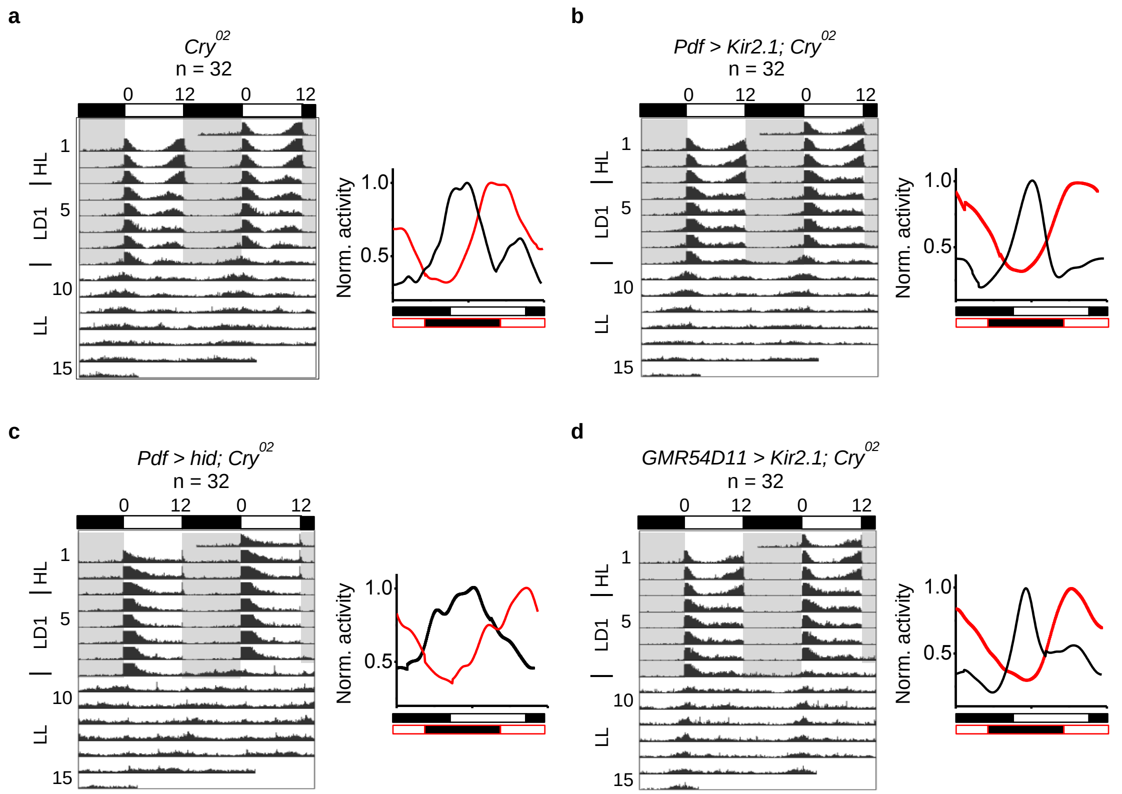

Supplementary Fig. 1. Photoentrainment of flies to LD1 cycles. (**a**) Averaged actogram of *Cry*^{02} flies. Left, actogram for *Cry*^{02} flies, which are firstly photoentrained by 3 high-light cycles (HL), followed by 5 dim-light cycles (LD1), and are then released to constant dim light (LL). Right, the phase difference (~8 hrs) in constant dim light between the flies with (from Fig. 1, red) and without (left panel, black) photoentrainment by the LD2 cycles with an 8-hr phase delay from the LD1 cycles. Normalized average activity is plotted for the first 4 dim LL cycles. The x-axis corresponds to the circadian time, with the same color for the corresponding activity trace. (**b**) *Pdf > kir2.1*; *Cry*^{02} flies. (**c**) *Pdf > hid*; *Cry*^{02} flies. (**d**) *GMR54D11 > kir2.1*; *Cry*^{02} flies.

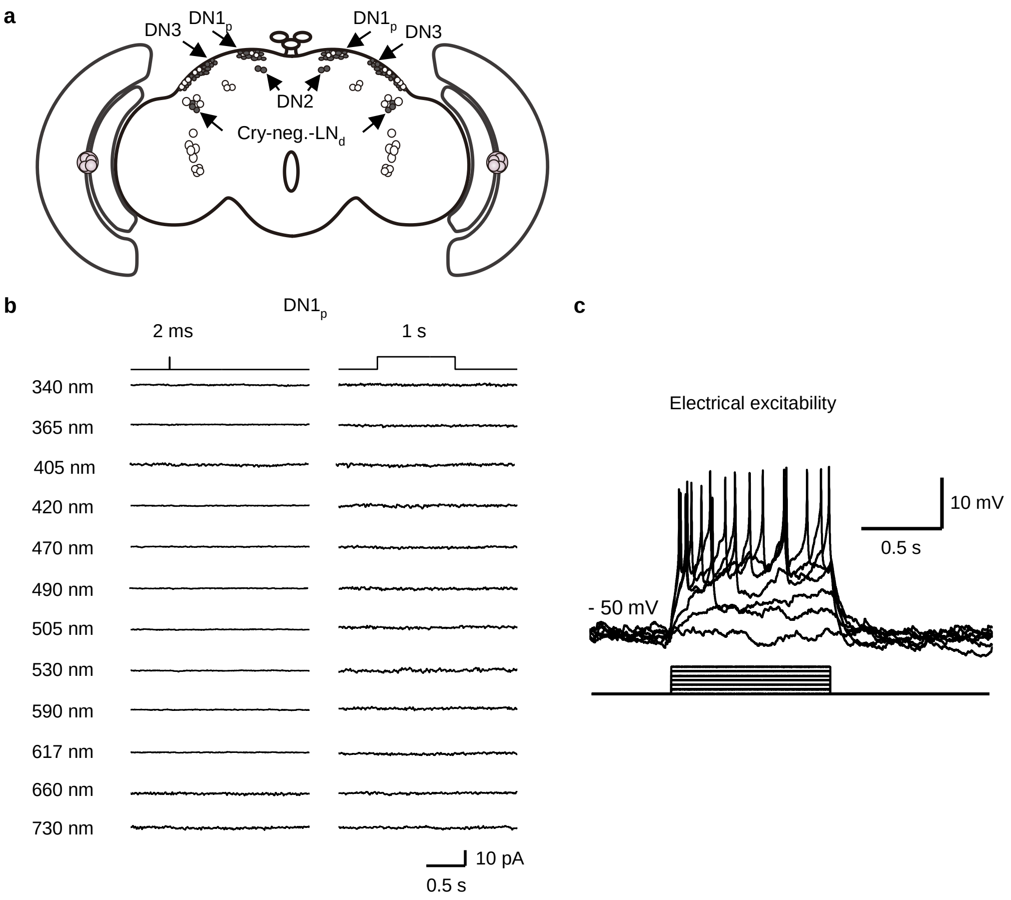

Supplementary Fig. 2. Light-insensitive central pacemaker neurons. (a) Schematic illustration of the types and location of light-insensitive DN1p, DN2, DN3, and Cry-negative LNd cells. **(b)** Representative recordings of light-insensitive pacemaker neurons (DN1p cells). Left, recordings (voltage clamp) to 2-ms flashes across all the wavelengths of 340, 365, 405, 420, 470, 490, 505, 530, 590, 617, 660, and 730 nm. Right, recordings to 1-s flashes. **(c)** Electrical excitability by current injections in DN1p cells (steps of 2 pA).

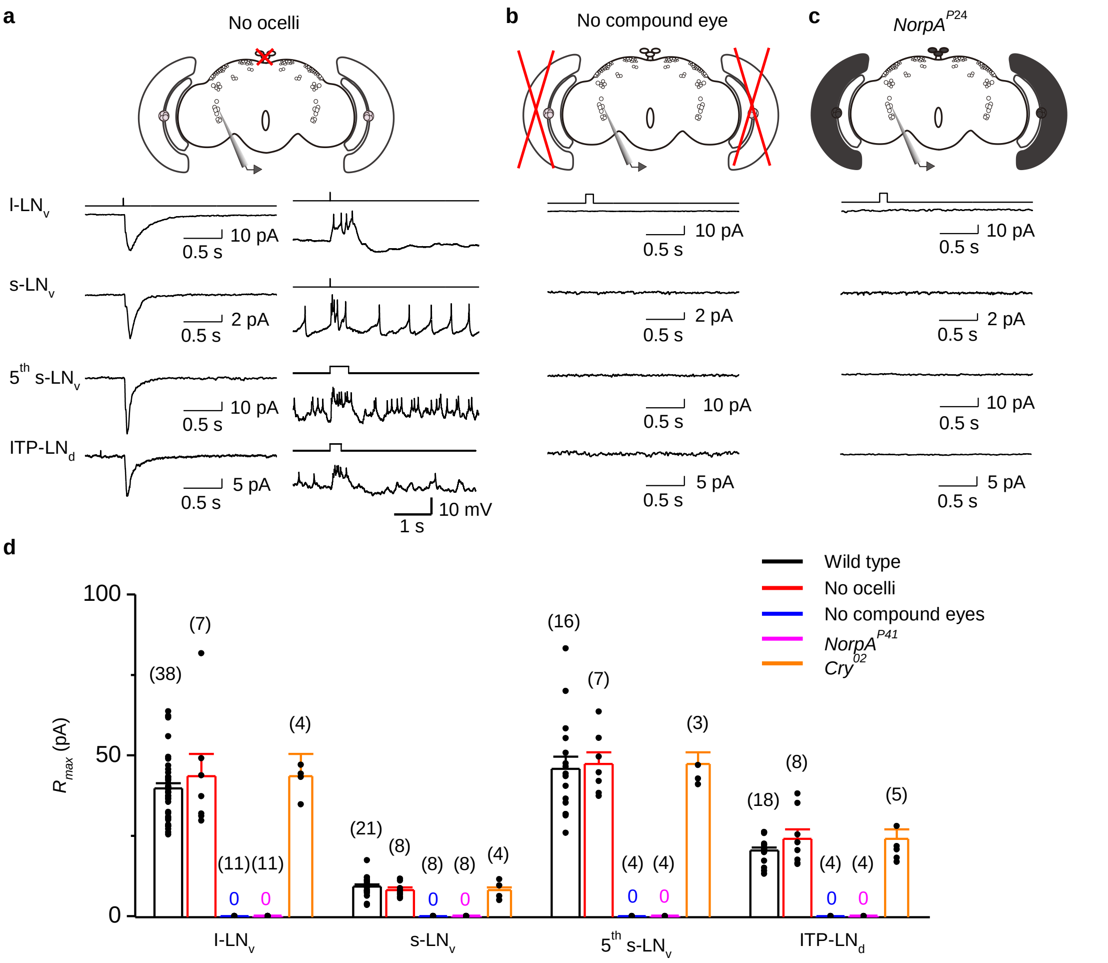

Supplementary Fig. 3. Eye inputs to central pacemaker. (a) Removal of three ocelli does not affect light responses of pacemaker neurons. **(b)** Removal of compound eyes abolishes light responses of pacemaker neurons. **(c)** Light responses are eliminated in pacemaker neurons of *NorpA*^{P24} flies. **(d)** Average response amplitudes of pacemaker neurons in flies as indicated. “0” indicates no responses at all. Cell numbers are indicated in brackets.

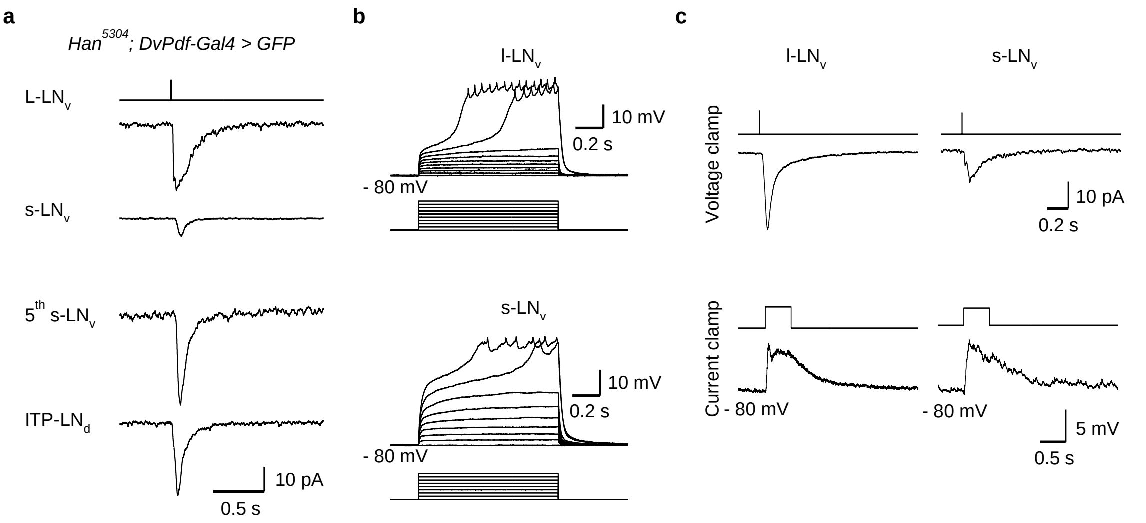

Supplementary Fig. 4. Central pacemaker neurons respond to light independently of one another. (a) Light responses remain intact in pacemaker neurons of *Pdfr* mutant flies. **(b)** Hyperpolarization and increased thresholds of action potential firing in l-LNvs (top) and s-LNvs (bottom) that express *Kir2.1* with *Pdf-LexA*. Resting membrane potentials are typically of -80 mV, compared to -45 mV in control flies. Large current injection (>140 pA) is required to trigger action potential firing, which is much larger than current responses (10-40 pA) induced by light in PDF-expressing LNvs. **(c)** Excitatory postsynaptic currents (EPSC, top) and potentials (EPSP, bottom). Light triggers the l-LNvs (left) and s-LNvs (right) that express *Kir2.1* to produce inward current but does not produce enough depolarization for spike generation.

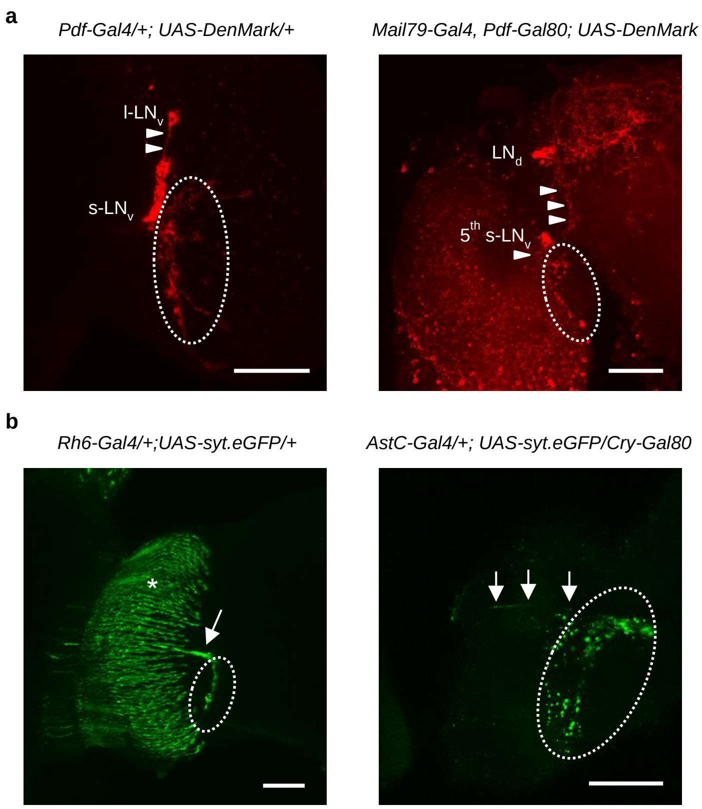

Supplementary Fig. 5. Identification of dendrites and axons by specific markers. **(a)** The arborization of clock neurons in accessary medulla are dendrites, identified by DenMark. Left, s-LNvs and l-LNvs. Right, the 5th s-LNv and ITP-LNd neurons. Arrow heads indicate the process that innervates to and then arborizes in accessary medulla. **(b)** The projections of H-B eyelet and *AstC* neuron to accessary medulla are axons, identified by syt.eGFP. Left, H-B eyelet photoreceptors. Right, *AstC* neuron. Arrows indicate the process that projects to accessary medulla. Dashed circles indicate the region of accessary medulla. * indicates the axons of R8 photoreceptors. Scale bar: 50 μm.

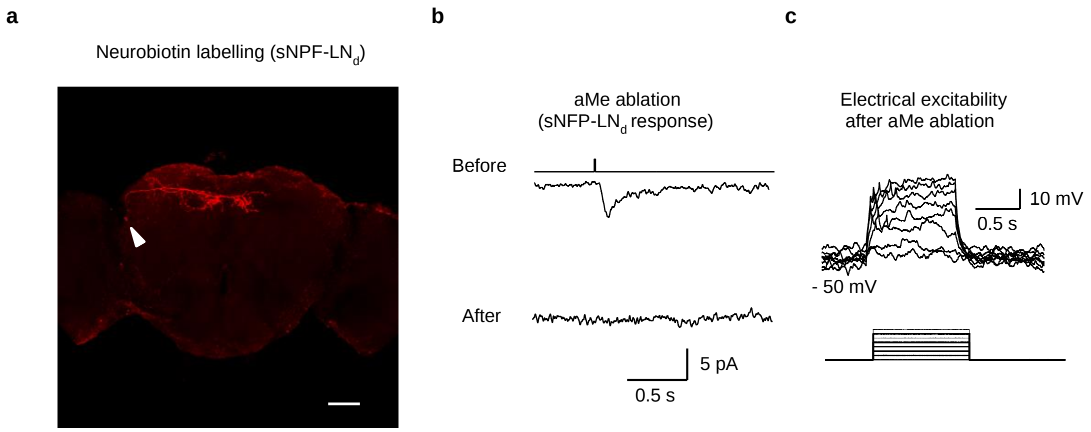

Supplementary Fig. 6. Light responses of sNPF-LNds depends on aMe.
(a) Neurobiotin labeling of sNPF-LNds. sNPF-LNds do not send dendrites to the aMe. Scale bar: 50 μm. Arrow head indicates the cell body of sNPF-LNd. **(b)** aMe is required for light sensitivity of sNPF-LNds. Light-induced electrical responses of the sNPF-LNd before (top) and after (bottom) laser ablation of the aMe. Light stimulation: 2 ms, 470 nm. **(c)** Electrical excitability of sNPF-LNds after laser ablation of aMe. Current steps: 5 pA.

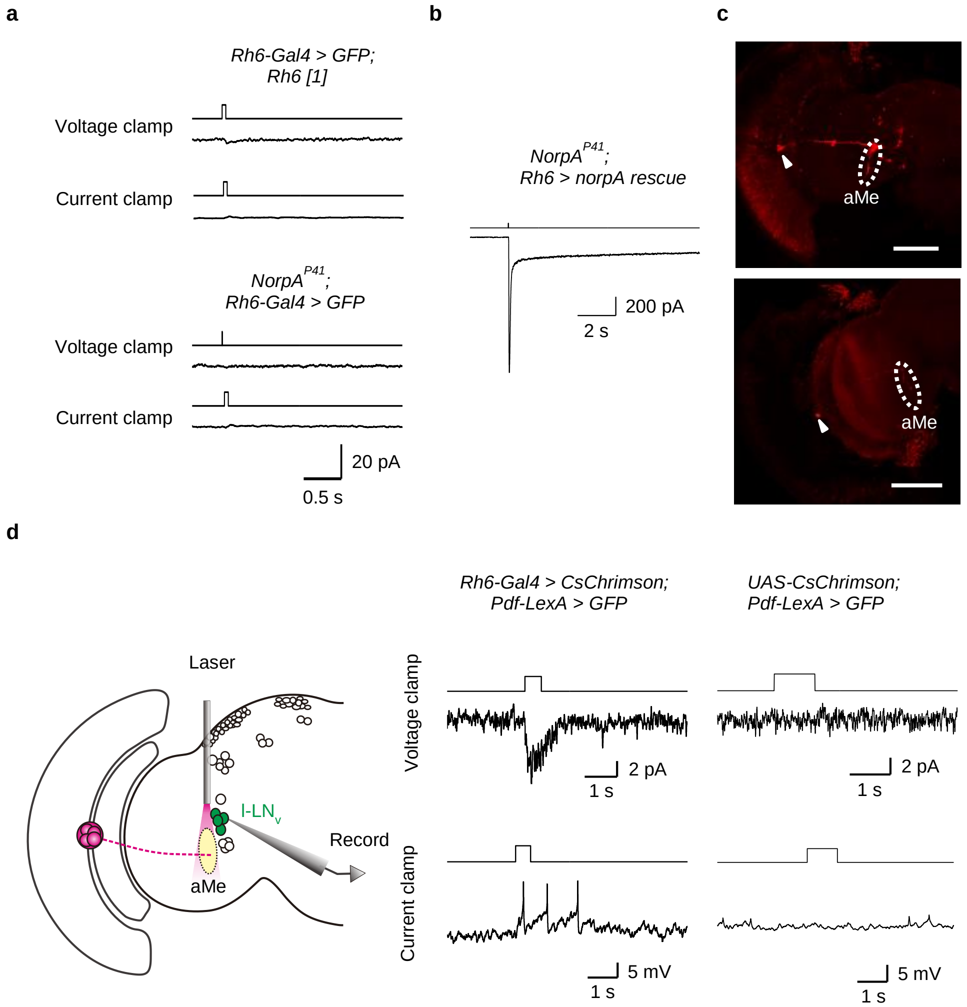

Supplementary Fig. 7. Visual signals from H-B eyelets to central pacemaker neurons. (a) Light responses of H-B eyelets are eliminated in *Rh6[1]* or $NorpA^{P41}$ mutant flies. (**b**) Light responses of H-B eyelets are rescued by expression of *norpA* in $NorpA^{P41}$ mutant flies **(c)** Neurobiotin injection to a single eyelet photoreceptor (arrowhead) in WT (top) and in $shakB^2$ flies (bottom) that lack gap junctions mediated by *shakB.neural* proteins. Scale bar: 50 µm. **(d)** H-B eyelets excite pacemaker neurons. Left, schematic illustration of simultaneous two-photon optogenetic activation of H-B eyelet axons in the aMe and patch clamp recordings of l-LNvs. Middle, current (top) and voltage (bottom) responses of the l-LNvs. Right, negative control of l-LNvs in the absence of *Rh6-Gal4*.

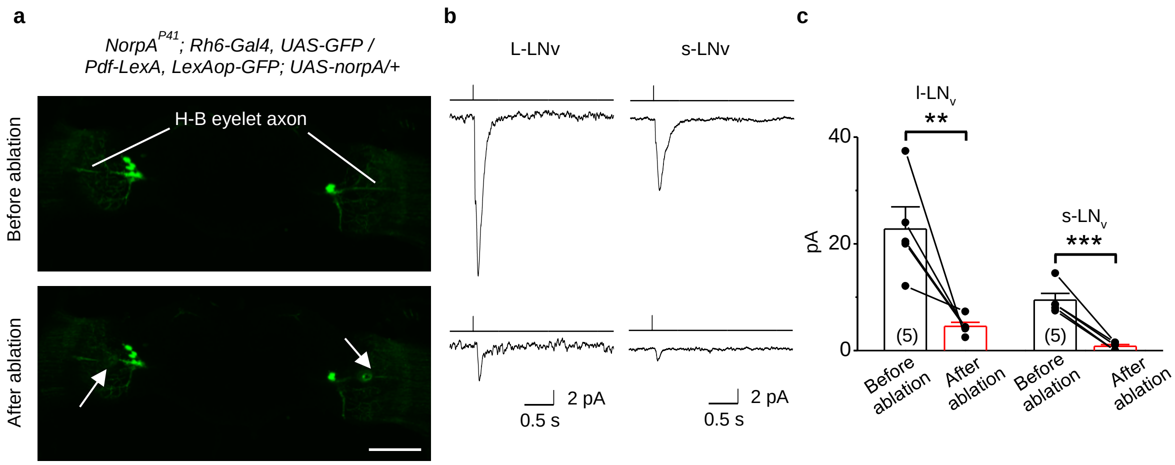

Supplementary Fig. 8. Laser ablation of H-B eyelet axons eliminates visual inputs from H-B eyelet to l-LNvs and s-LNvs. (a) GFP-labeled axons of H-B eyelet before (top) and after (bottom) laser ablation in *Rh6-Gal4*-driven *norpA* rescue flies. Arrows indicate the ablated regions. Scale bar: 50 µm. **(b)** Light-induced electrical responses of l-LNvs (left) and s-LNvs (right) before (top) and after (bottom) laser ablation of H-B eyelet axons. Light stimulation: 2 ms, 470 nm. **(c)** Collective data in (b). n = 5 and 5 for l-LNv and s-LNv, respectively; error bars represent SEM; ** $P < 0.01$; *** $P < 0.001$.

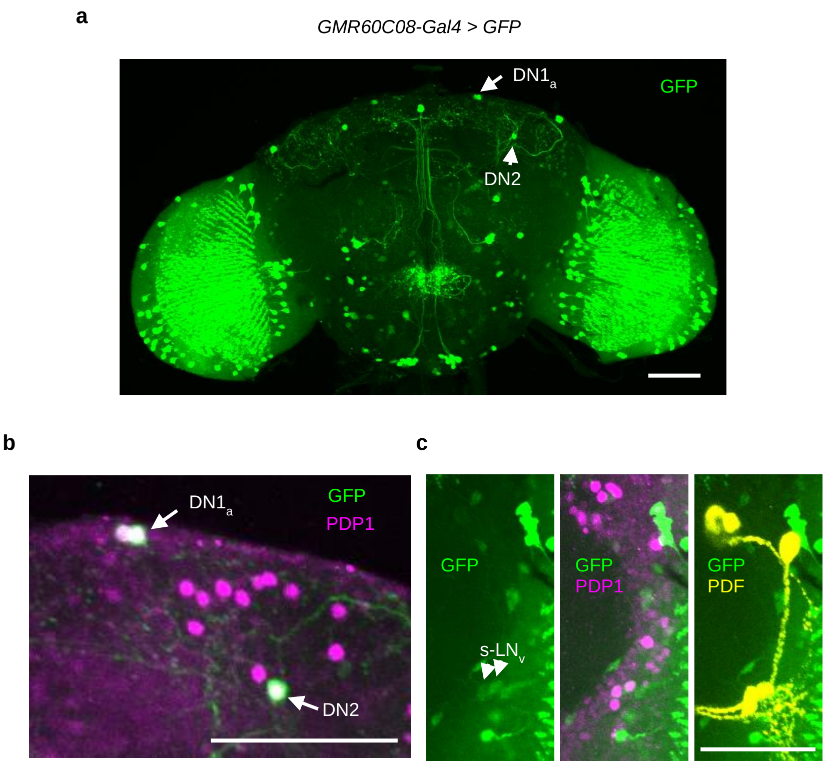

Supplementary Fig. 9. *GMR60C08-Gal4* labels DN1a. (**a**) *GMR60C08-Gal4* labels two DN1a, one DN2 and two s-LN$_v$ neurons. GFP immunostaining in the whole brain. (**b**) GFP and *PAR* domain protein 1 (*PDP1*) double immunostaining in DN groups. (**c**) GFP, PDP1 and PDF triple immunostaining in LN groups. Scale bar: 50 μm.

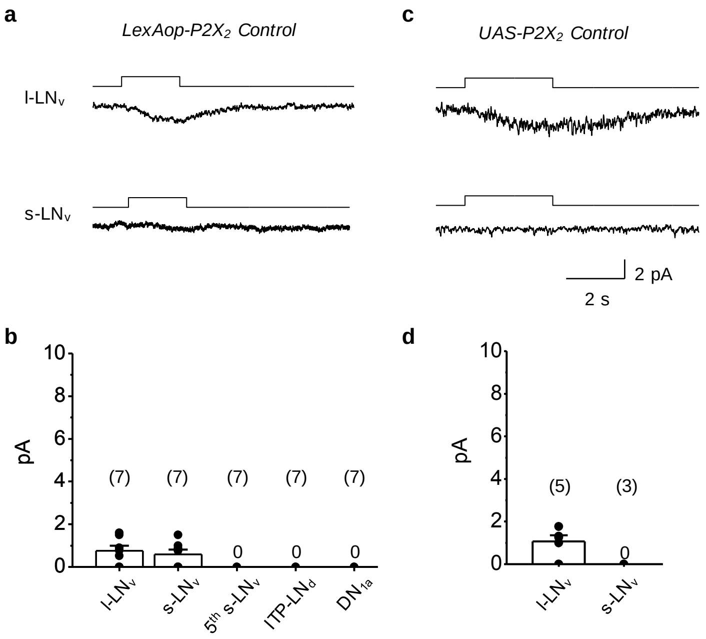

Supplementary Fig. 10. Leaky $P2X_2$ expression by *LexAop-$P2X_2$* and *UAS-$P2X_2$* in the absence of corresponding drivers. (a) Representative responses of l-LNv (top) and s-LNv (bottom) to leaky $P2X_2$ expression by *LexAop-$P2X_2$*. ATP: 2.5 mM, 2 s. **(b)** Collective data for *LexAop-$P2X_2$*. **(c)** Representative responses of l-LNv (top) and s-LNv (bottom) to leaky $P2X_2$ expression by *UAS-$P2X_2$*. ATP: 2.5 mM, 3 s. **(d)** Collective data for *UAS-$P2X_2$*.

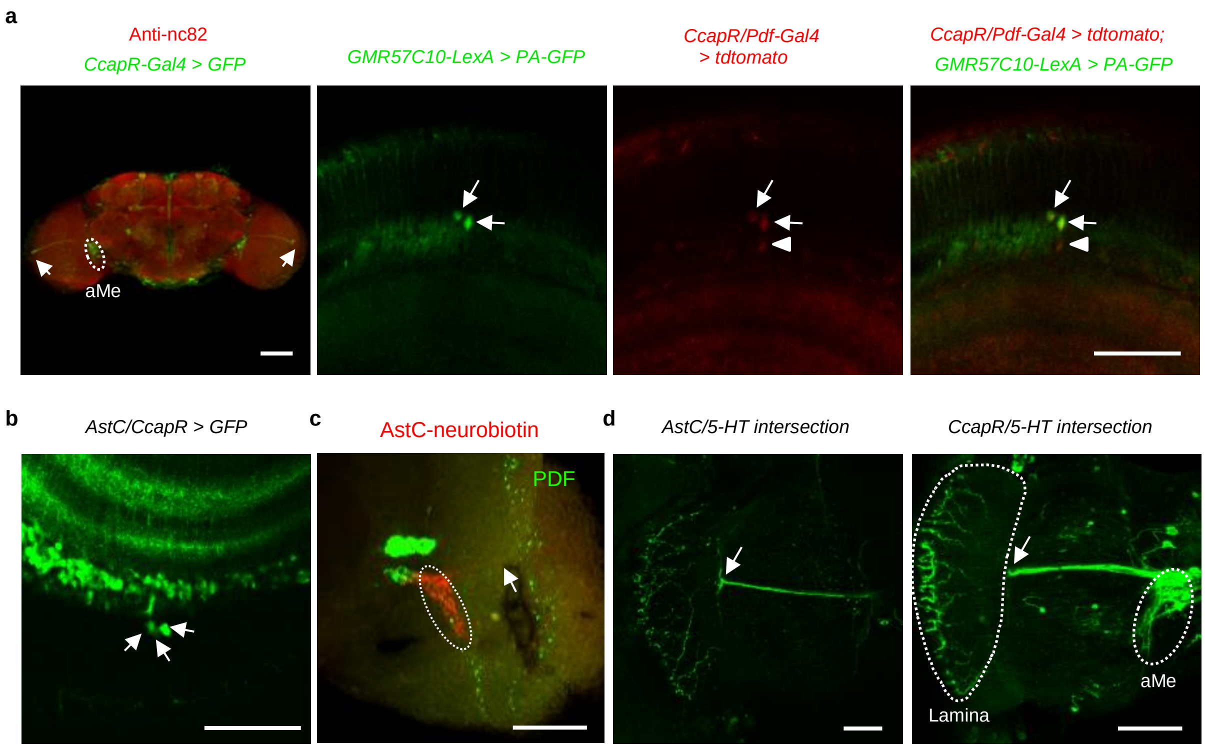

Supplementary Fig. 11. Interneurons connecting compound eyes and pacemaker neurons. (a) *CcapR-Gal4* labels three interneurons, with two of them labelled by PA-GFP. Arrows indicates the cell bodies, and arrowheads indicate the non-overlapping cell body. Scale bar: 50 µm. **(b)** Overlay of interneuron labelling by *AstC-Gal4* (one cell) and *CcapR-Gal4* (three cells). Scale bar: 50 µm. **(c)** *AstC* interneuron projects to aMe. Overlay of neurobiotin labelling of the *AstC* interneuron and PDF immunostaining. Dashed circle indicates the aMe, and arrow indicates the *AstC* axon. Scale bar: 50 µm. **(d)** Dendritic arborization of *AstC* and *CcapR* interneurons in lamina. Scale bar: 50 µm.

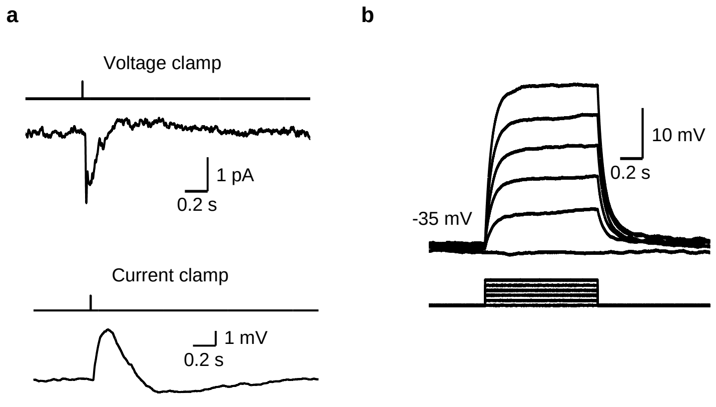

Supplementary Fig. 12. Electrical response properties of the *AstC* interneuron.
(a) Light responses of the *AstC* interneuron. Light induced an inward current (top) that produces graded depolarization (bottom). **(b)** Current injections do not trigger action potential firing in the *AstC* interneurons (steps of 5 pA).

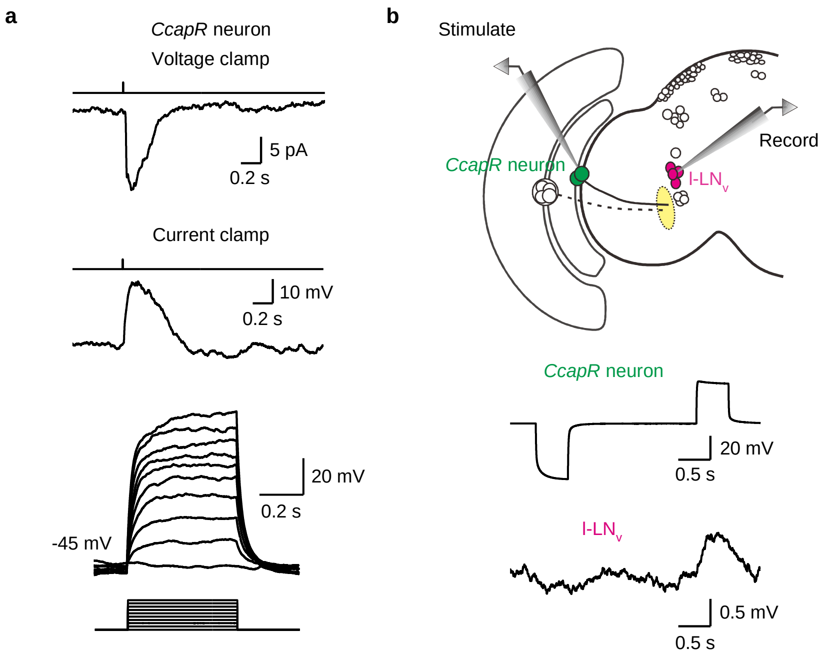

Supplementary Fig. 13. *CcapR* neurons relay light signals from compound eyes to pacemaker neurons. (a) Light responses of the *CcapR* interneuron. Top, light induces graded depolarization of *CcapR* interneurons. Bottom, current injections do not trigger action potential firing in *CcapR* interneurons (steps of 10 pA). **(b)** *CcapR* interneurons directly excite l-LNvs. Top, illustration of dual patch-clamp recordings between the *CcapR* interneuron and l-LNvs. Bottom, electrical depolarization of the *CcapR* interneuron excites the l-LNv neuron.

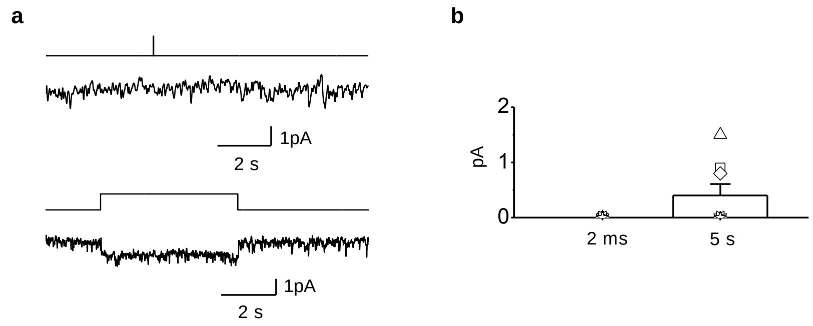

Supplementary Fig. 14. Eye-independent responses of l-LNvs to intense and long light stimuli. (a) Top, 2-ms light flash of combined four LEDs with peak wavelengths of 365, 490, 617 and 660 nm (each at its maximal intensity) does not trigger any electrical responses in l-LNv of the eyeless brain preparation. Bottom, 5-s light step of the same set of four LEDs at maximal intensities triggers a tiny response of ~ 1 pA in the same l-LNv as in the top. **(b)** Collective data in (**a**).

Supplementary Table 1: Genotypes used in this study

**Figure**	**Genotype**
Figure. 1a	*Cry*^{02}
Figure. 1b	*NorpA*^{P41}*;;Cry*^{02}
Figure. 1c	*Pdf-Gal4/UAS-Kir2.1; Cry*^{02}
Figure. 1d	*Pdf-Gal4, UAS-hid; Cry*^{02}
Figure. 1e	*UAS-Kir2.1/+; GMR54D11-Gal4,Cry*^{01}*/Cry*^{02}
Figure. 2a	*Clk856-Gal4, UAS-mCD8-GFP*
Figure. 2b	*C929-Gal4, UAS-mCD8-GFP*
Figure. 2c	*C929-Gal4; UAS-mCD8-GFP*<br>*R6-Gal4; UAS-mCD8-GFP*<br>*GMR54D11-Gal4, UAS-mCD8-GFP*<br>*Mai179-Gal4, Pdf-Gal80; UAS-mCD8-GFP*<br>*Cry39-Gal4, UAS-mCD8-GFP*<br>*Clk9M-Gal4, UAS-mCD8-GFP*<br>*UAS-mCD8-GFP; GMR60C08-Gal4*<br>*UAS-mCD8-GFP; Clk4.1M-Gal4*<br>*Clk856-Gal4, UAS-mCD8-GFP*
Figure. 3a	*Mai179-Gal4, UAS-mCD8-GFP; Pdf*^{01}<br>*Pdf-LexA /+;GMR54D11-Gal4,UAS-GFP/LexAop2-Kir2.1-GFP*
Figure. 3b	*DvPdf-LexA/+; LexAop2-GFP/+*<br>*Pdf-Gal4, UAS-hid/DvPdf-LexA; LexAop2-GFP/+*
Figure. 3c	*Pdf-LexA, LexAop2-GFP/UAS-Kir2.1; GMR54D11-Gal4/+*
Figure. 3d	*Cry39-Gal4, UAS-mCD8-GFP/DvPdf-LexA; LexAop2-Kir2.1-GFP/+*
Figure. 4a	*Clk856-Gal4, UAS-mCD8-GFP*
Figure. 4c	*DvPdf-Gal4, UAS-mCD8-GFP*
Figure. 5a	*Mai179-Gal4, Pdf-Gal80; UAS-mCD8-GFP*
Figure. 5b	*Pdf-LexA/Mai179-Gal4, UAS-mCD8-GFP; LexAop2-rpr/+*
Figure. 5c	*Pdf-LexA/Mai179-Gal4, UAS-mCD8-GFP; LexAop-P2X*$_2$*/+*
Figure. 5d, e	*Mai179-Gal4, Pdf-Gal80; UAS-mCD8-GFP*
Figure. 5f	*DvPdf-LexA/Mai179-Gal4, UAS-mCD8-GFP; LexAop2-Kir2.1-GFP/+*
Figure. 6b	*Rh6-Gal4, UAS-mCD8-GFP*
Figure. 6c	*DvPdf-Gal4, UAS-mCD8-GFP*<br>*DvPdf-Gal4, UAS-mCD8-GFP; Rh6[1]*<br>*NorpA*^{P41}*; DvPdf-Gal4, UAS-mCD8-GFP*<br>*NorpA*^{P41}*; DvPdf-LexA, LexAop2-GCaMP6f/Rh6-Gal4; UAS-norpA/+*
Figure. 6d	*Rh6-LexA, LexAop-GFP11/C929-Gal4; UAS-GFP1-10/+*<br>*Rh6-LexA, LexAop-GFP11/R6-Gal4; UAS-GFP1-10/+*<br>*Rh6-LexA, LexAop-GFP11/Mai179-Gal4, Pdf-Gal80; UAS-GFP1-10/+*<br>*Rh6-LexA, LexAop-GFP11/Pdf-Gal80; UAS-GFP1-10/GMR60C08-Gal4*

Figure. 6e	*Rh6-LexA/Cry39-Gal4; UAS-GCaMP6m, LexAop-$P2X_2$/+*
Figure. 7b	*DvPdf-Gal4, UAS-mCD8-GFP*<br>*DvPdf-Gal4, UAS-mCD8-GFP; NinaE^{I17}*<br>*NorpA^{P41}; DvPdf-Gal4, UAS-mCD8-GFP*<br>*NorpA^{P41}; DvPdf-LexA, LexAop2-GCaMP6f/ +; UAS-norpA/ Rh1-Gal4*
Figure. 7c	*Pdf-Gal4, UAS-tdtomato/GMR57C10-LexA; LexAop2-sPA-GFP/+*
Figure. 7d	*AstC-Gal4/UAS-mCD8-GFP*<br>*GMR57C10-LexA/AstC-Gal4; LexAop2-sPA-GFP/Pdf-Gal4, UAS-tdtomato*
Figure. 7e	*Pdf-LexA, LexAop-GFP11/AstC-Gal4; UAS-GFP1-10/Cry-Gal80*
Figure. 7f	*AstC-Gal4/Pdf-LexA, LexAop2-GFP; UAS-$P2X_2$/Cry-Gal80*
Figure. 7g	*AstC-Gal4/Pdf-Gal4,UAS-mCD8-GFP; Rh6[1]*
Supplementary Fig. 1a	*Cry^{02}*
Supplementary Fig. 1b	*Pdf-Gal4/UAS-Kir2.1; Cry^{02}*
Supplementary Fig. 1c	*Pdf-Gal4, UAS-hid; Cry^{02}*
Supplementary Fig. 1d	*UAS-Kir2.1/+; GMR54D11-Gal4,Cry^{01}/Cry^{02}*
Supplementary Fig. 2b,c	*UAS-mCD8-GFP; Clk4.1M-Gal4*
Supplementary Fig. 3a,b	*DvPdf-Gal4, UAS-mCD8-GFP*
Supplementary Fig. 3c	*NorpA^{P24}; DvPdf-Gal4, UAS-mCD8-GFP*
Supplementary Fig. 4a	*Han^{5304}; DvPdf-Gal4, UAS-mCD8-GFP*
Supplementary Fig. 4b, c	*Pdf-LexA/+; LexAop2-Kir2.1-GFP/+*
Supplementary Fig. 5a	*Pdf-Gal4/+; UAS-DenMark/+*<br>*Mai179-Gal4, Pdf-Gal80; UAS-DenMark*
Supplementary Fig. 5b	*Rh6-Gal4/+; UAS-syt.eGFP/+*<br>*AstC-Gal4/+; UAS-syt.eGFP/Cry-Gal80*
Supplementary Fig. 6b, c	*Mai179-Gal4, UAS-mCD8-GFP*
Supplementary Fig. 7a	*Rh6-Gal4, UAS-mCD8-GFP; Rh6[1]*<br>*NorpA^{P41}; Rh6-Gal4, UAS-mCD8-GFP*
Supplementary Fig. 7b	*NorpA^{P41}; Rh6-Gal4, UAS-mCD8-GFP/+; UAS-norpA/+*
Supplementary Fig. 7c	*Rh6-Gal4, UAS-mCD8-GFP*<br>*shakB^{2}; Rh6-Gal4, UAS-mCD8-GFP*
Supplementary Fig. 7d	*NorpA^{P41}; Pdf-LexA, LexAop2-myrGFP/Rh6-Gal4; UAS-CsChrimson/+*<br>*NorpA^{P41}; Pdf-LexA, LexAop2-myrGFP/+; UAS-CsChrimson/+*
Supplementary Fig. 8	*NorpA^{P41}; Pdf-LexA, LexAop2-GFP/Rh6-Gal4, UAS-GFP; UAS-norpA/+*
Supplementary Fig. 9a-c	*UAS-GFPS65T/+; GMR60C08-Gal4/+*
Supplementary Fig. 10a, b	*Cry39-Gal4/+; UAS-GCaMP6m, LexAop-$P2X_2$/+*
Supplementary Fig. 10c, d	*Pdf-LexA, LexAop2-GCaMP6f/+; UAS- $P2X_2$/+*
Supplementary Fig. 11a	*Pdf-Gal4, UAS-tdtomato/GMR57C10-LexA; CcapR-Gal4/LexAop2-sPA-GFP*<br>*CcapR-Gal4/UAS-mCD8-GFP*
Supplementary Fig. 11b	*AstC-Gal4/+; CcapR-Gal4/UAS-mCD8-GFP*
Supplementary Fig. 11d	*AstC-Gal4/+; Trh-FLP, UAS-FRT-Stop-FRT-GFP/+*

	Trh-FLP, UAS-FRT-Stop-FRT-GFP/CcapR-Gal4
Supplementary Fig. 12a,b	*AstC-Gal4; UAS-mCD8-GFP*
Supplementary Fig. 13a	*UAS-mCD8-GFP; CcapR-Gal4*
Supplementary Fig. 13b	*Pdf-Gal4, UAS-mCD8-GFP/+; CcapR-Gal4/+*
Supplementary Fig. 14a, b	*Pdf-Gal4, UAS-mCD8-GFP;+*
